# Navigating Lipid Nanostructure Design Space Through Continuous Microfluidic Automation

**DOI:** 10.1002/adma.74165

**Published:** 2026-07-27

**Authors:** Bradley Diggines, Marcus Fletcher, Manuel Bibrowski, Karnyart Samnuan, Rongjun Chen, David J. Peeler, Amjad Abouselo, Morag R. Hunter, Molly M. Stevens, Nicholas J. Brooks, Yuval Elani

**Affiliations:** ^1^ Department of Chemical Engineering Imperial College London London UK; ^2^ Department of Chemistry Imperial College London London UK; ^3^ Department of Materials Department of Bioengineering and Institute for Biomedical Engineering Imperial College London London UK; ^4^ Department of Physiology Anatomy and Genetics Department of Engineering Science Kavli Institute for Nanoscience Discovery University of Oxford Oxford UK; ^5^ Department of Bioengineering University of Oregon Eugene Oregon USA; ^6^ Early Product Development & Manufacturing Pharmaceutical Sciences R&D AstraZeneca Macclesfield UK; ^7^ Centre For Genomics Research Discovery Sciences R&D AstraZeneca Cambridge UK

**Keywords:** automation, bottleneck, computer science, microfluidics, nanomaterials, nanoscopic scale, nanotechnology, particle, throughput, workflow

## Abstract

The versatility of nanoscale lipid particles has positioned them as the scaffold of choice for biomedical delivery, synthetic membrane engineering, and fundamental biophysical exploration. Across these fields, rational particle design has become a major bottleneck. Lipid composition, stoichiometry, size, morphology, phase behavior, and biophysical properties combine into an enormous, high‑dimensional space that is inherently difficult to explore, making conventional optimization approaches slow and limited. Identifying optimal lipid compositions in this vast space necessitates high‐throughput screening based on efficient low‐cost automation. Here we introduce a high‐throughput microfluidic platform for rapid screening of lipid nanoparticles, offering precise, programmable control over composition and morphology. The system integrates on‐chip microfluidic metering of lipid stocks with continuous particle self‐assembly, followed by robotic collection into 96‐well plates. This workflow enables the generation of over 200 unique formulations per hour, delivering a several‐orders‐of‐magnitude increase in throughput relative to conventional approaches. We apply the platform to systematically map biophysical space at unprecedented resolution, validate compositional trends in transfection efficiency, and identify non‐lamellar formulations with optimal functional performance. By coupling scalable synthesis with automated screening, we expect this platform to provide a robust foundation for data‐driven and AI‐integrated discovery of self‐assembled nanomaterials including lipid, polymeric, and biomolecular assemblies.

## Graphical abstract

LipidXplorer is a novel microfluidic platform which enables rapid navigation of lipid particle design space for high‐resolution phase mapping, lipid nanoparticle optimization, membrane biophysics, and functional nanomaterial discovery. It combines programmable composition control, continuous nanoparticle formation, and automated well‐plate collection to produce more than 200 precisely defined particle formulations per hour across diverse applications.

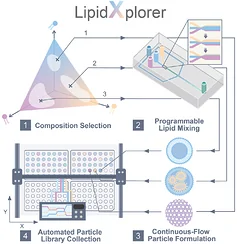

## 1 Introduction

Nanoscale lipid particles—including, but not limited to, lipid‐nucleic acid nanoparticles [1], liposomes [2] and non‐lamellar assemblies such as cubosomes [3] and hexosomes [4]—are a versatile class of technologies that bridge multiple biological disciplines, encompassing applications in drug delivery [5], biosensing [6, 7], synthetic biology [8], and fundamental biophysical research [9, 10]. Owing to their tunable morphology, ease of assembly, and compatibility with biomolecules, they provide an ideal scaffold for encapsulation, functionalization and intermolecular networking [11]. Lipid‐based nanoparticles are now ubiquitous across diverse applications, serving not only as clinically approved delivery vehicles for therapeutic cargos such as mRNA [1], but also as programmable biochemical reactors [12] enabling synthetic signaling [13] and catalytic pathways [14], and as model systems of biological membranes for probing protein insertion [15] and lateral domain formation [16].

Fine‐tuning the lipid composition of nanoparticles is central to optimizing function, as membrane properties are acutely sensitive to stoichiometry. Composition‐dependent effects underpin a wide range of design challenges: for example, efficient endosomal escape of mRNA lipid nanoparticles exploits transitions from lamellar to hexagonal phases [17, 18], while successful membrane protein insertion depends critically on identifying bilayers with hydrophobic thickness and mechanical properties matched to the target protein [19, 20]. More broadly, subtle changes in lipid composition can alter fundamental membrane parameters such as curvature, phase behavior, and particle size, thereby reshaping processes such as encapsulation, fusion, and biomolecular interactions. While powerful, this sensitivity gives rise to vast, high‐dimensional design spaces in which identifying optimal compositions remains a formidable challenge, motivating the need for approaches capable of exploring lipid formulation space at unprecedented scale.

Effective exploration of these vast and complex nanoparticle design spaces demands throughput beyond the limits of conventional, human‐led rational design and experimentation. This scale is increasingly achievable through automatable microfluidic platforms, which enable rapid, reproducible synthesis and screening of thousands of distinct formulations [21]. The value of microfluidic screening has already been demonstrated in adjacent soft‐matter and protein systems, where platforms such as PhaseScan [22] and PhaseXplorer [23] have enabled high‐throughput mapping of biomolecular condensate phase behavior. The resulting large, high‐quality datasets spanning composition space create opportunities for the direct application of modern artificial intelligence (AI) and machine‐learning (ML) approaches, which can capture intricate composition–function relationships that remain inaccessible to traditional physics‐based models [24]. This, in turn, enables iterative cycles of large‐scale library generation and AI‐driven optimization, an approach that has proven transformative in related fields such as small‐molecule drug discovery and materials science.

Unfortunately, lipid particle synthesis remains poorly aligned with modern high‐throughput discovery workflows. Conventional synthesis methods, such as extrusion [25], yield high‐quality particles, but are slow and labor‐intensive. In contrast, robotic bulk‐mixing approaches [26] are fast but lack control over morphology, leading to substantial variability in particle size and polydispersity. Although microfluidic platforms bring improved size control, most existing systems are lipid nanoparticle (LNP)‐specific and are either restricted to single‐composition batch formulation [27], rely on costly, proprietary automation with long per‐sample processing times (∼3.75 min per sample [28]; 6 h per 96 well plate), or have variability in compositional control [29]. Collectively, these technical and accessibility constraints have hindered data‐driven exploration of compositional space beyond the context of industrial pharmaceutical lipid nanoparticle research.

To address this challenge, we present LipidXplorer—a modular microfluidic–robotic pipeline for the continuous synthesis and automated plating of large libraries of lipid particles. Continuous microfluidic lipid selection and formulation generate discrete and precise particle compositions at rates orders of magnitude faster than conventional methods. These particles are collected directly into standard microtiter plates, enabling seamless integration with existing high‐throughput plate‐based screening workflows [30] for key characterization techniques (cell‐culture assays, dynamic light scattering [31], fluorescence, and x‐ray diffraction, for example). Having established the system, we validate operational performance and demonstrate its utility for high‐resolution biophysical mapping, transfection optimization and identification of peak‐performing compositions. Designed to align with existing microfluidic expertise, LipidXplorer lowers the barrier to high‐throughput experimentation across all aspects of lipid nanotechnology. By enabling rapid, systematic navigation of complex compositional landscapes, the platform provides a practical route to uncovering structure–function relationships that are otherwise inaccessible using conventional formulation approaches.

## 2 Results and Discussions

### 2.1 Operating Principles of the LipidXplorer Platform

**FIGURE 1:**
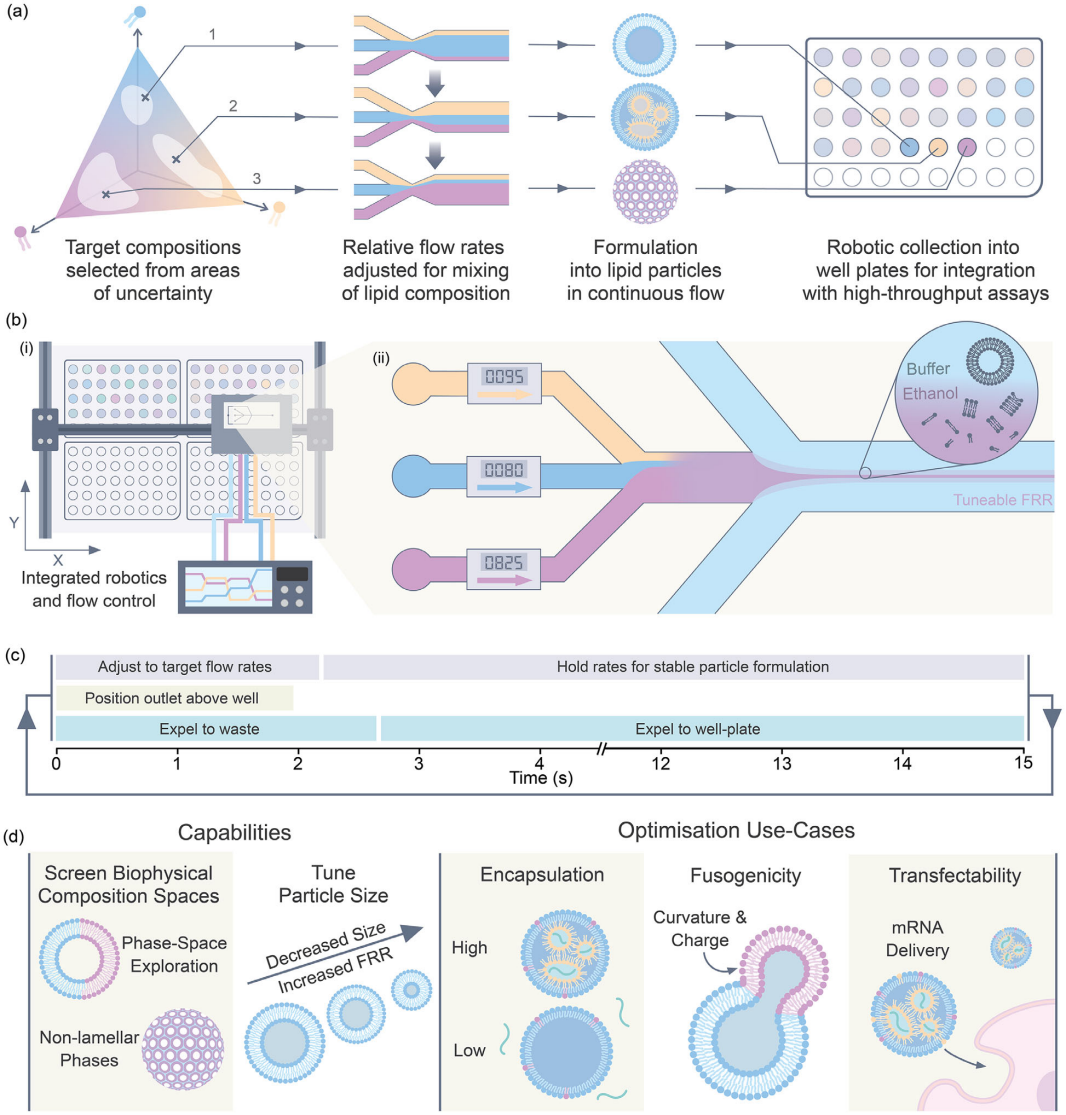
Fundamentals of LipidXplorer. (a) Screening pipeline: target compositions are first selected from unexplored or promising regions of compositional space and generated by continuous microfluidic mixing of lipid stocks, followed by particle formulation and automated collection into 96‐well plates for downstream analysis. (b) Programmed flow control, with active selection and mixing of lipid stocks before particle formulation, integrated with robotic handling, enables continuous production of large compositional libraries with fine control over particle composition and size. (c) Typical process timeline for a single formulation, in which flow rates are adjusted to achieve the target composition, allowed to stabilize, and then collected into a well plate for a defined volume. (d) Representative capabilities and application areas of the platform we demonstrate across a range of lipid‐based nanotechnologies.

The LipidXplorer platform is a microfluidic formulation system that selects discrete lipid compositions from a ternary space, formulates them into nanoscale particles, and sequentially collects them into screening‐compatible 96‐well plates (Figure 1a). As in conventional microfluidic nanoparticle formulation, thin streams of lipid‐containing organic solvent flow alongside an aqueous buffer, where controlled diffusion produces uniformly sized lipid nanoparticles. LipidXplorer builds upon this principle with coordinated microfluidic and robotic steps to feed selected lipids into the particle assembly stream before collecting the stoichiometrically accurate nanoparticles into a compositional library (Figures 1bi, ii and S1a). All of this is achieved through the strategic use of conventional microfluidic technologies and automation hardware, enabling broad accessibility and straightforward adaptation to alternative microfluidic devices and amphiphilic systems.

Core to the platform is the microfluidic selection of lipids from the stock reservoirs. For each composition (specified by human input or ML guidance), three lipid stock solutions mix in continuous flow to produce a homogeneous solution at a concentration proportional to the relative flow rates of each stream [32] (Equation S1), calculated by an integrated algorithm. Flow is pressure‐driven to achieve millisecond response times, with flow rates continuously monitored using specially calibrated flow sensors. Flow rates are regulated by a custom flow‐pressure algorithm (Equation S2) ensuring rapid changes while maintaining accuracy and stability. Because each composition is generated deterministically from programmed flow rates, collected particles can be compositionally assigned without fluorescent barcoding, unlike many microfluidic compositional screening systems. This control system also enables more flexible and resilient flow, able to adapt to changes in resistance induced by chip‐to‐chip or compositional variations in a way that numerical predictive methods [29] cannot. The lipid stock mixture proceeds to meet an aqueous formulation buffer (Figure 1bii) at a 45° microfluidic hydrodynamic focusing (MHF) [33] junction (or alternative advective mixing geometry). At the interface between the two phases, the nanoparticles self‐assemble with their composition equal to that of the set lipid stock. Given the integration of these two steps, particles with any composition in a vast ternary space may be produced simply by setting the relative flow rates (i.e., if the flow rate of lipid A is twice that of lipid B, the resulting particles will have an A:B stoichiometry of 2:1). Such an approach allows the unique selection of non‐sequential compositions, which can be dictated quickly in real‐time. It is also advantageous logistically—removing the requirement for specialized dispensing systems or ethanol evaporation concerns when working with lipid stocks. The flow‐control architecture is inherently modular and scalable: input lines can be added or removed to suit a given formulation or microfluidic device, allowing the combination of more than three lipid stock components. In addition to composition, the size may also be tuned by changing the flow rate ratio between the aqueous and solvent streams as previously established [34], with larger ratios resulting in smaller particles and vice versa.

The integration of these two microfluidic processes produces a continuous stream of lipid particles with a prescribed composition; however, effective downstream use requires their organization into screening‐compatible 96‐well plate libraries. This is achieved through an integrated robotic sorting system (Figure 1bi), which positions the microfluidic outlet above individual wells in accordance with the control algorithm (Figures 1c and S1b). Because the system operates under continuous flow, we designed a novel, contactless flow‐control valve to divert waste and ensure that only particles produced under the correct flow conditions are collected (Figure S1c). Importantly, this valve does not disrupt flow stability between collection steps, allowing steady‐state conditions to be reached and maintained as collection begins. Together, these integrated microfluidic and robotic components form a streamlined pipeline for producing lipid particle composition libraries by combining well‐established technologies. This approach enables systematic screening across coupled composition–size spaces, facilitating exploration of a broad range of biomedical and biophysical properties, several of which are illustrated in Figure 1d.

### 2.2 Validation of Compositional Control and Platform Performance

#### 2.2.1 Compositional Control and Operation

**FIGURE 2:**
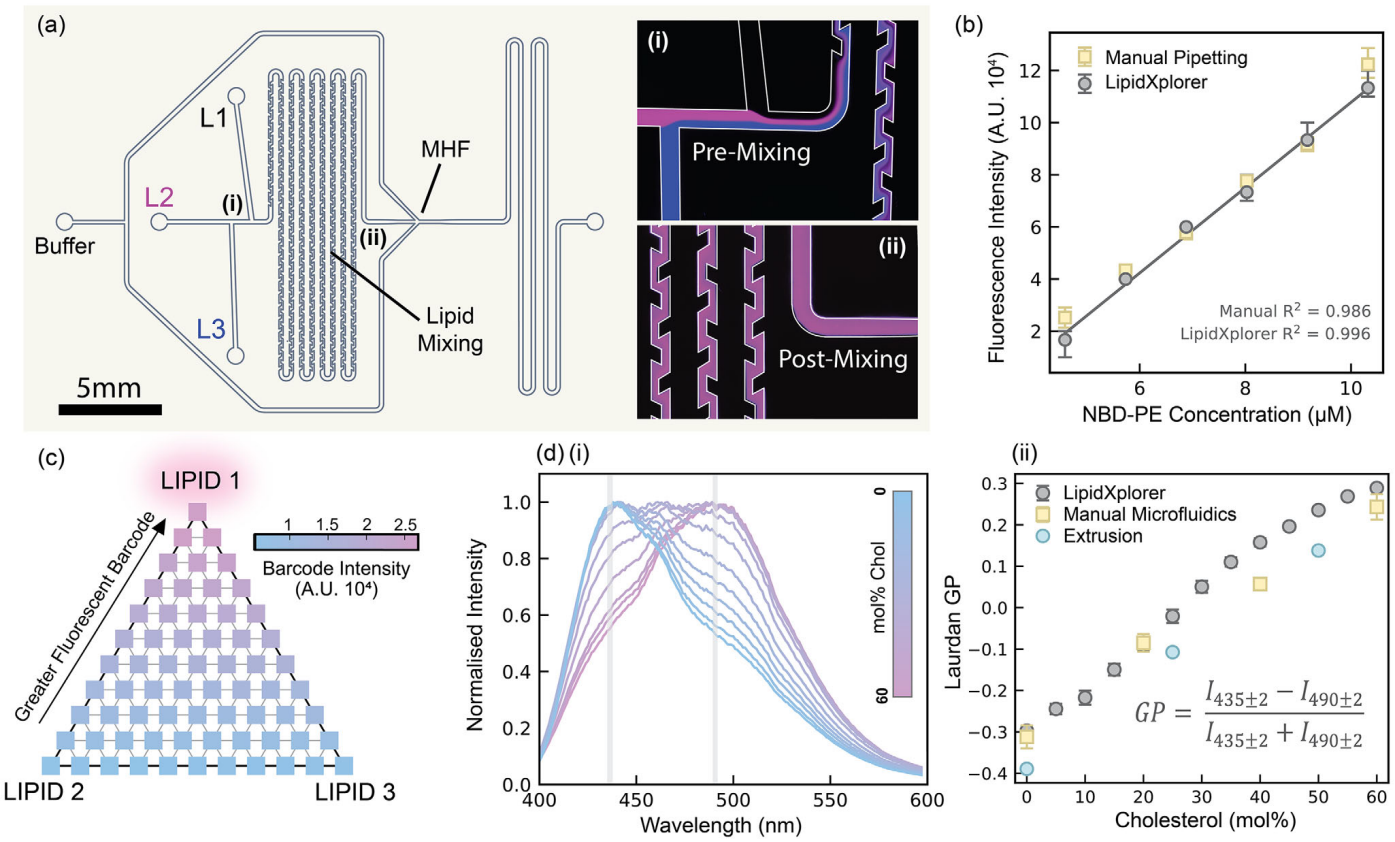
Validation of the LipidXplorer platform (a) Efficient mixing of lipid stocks within the advective channel geometry is achieved upstream of the microfluidic hydrodynamic focusing (MHF) junction (point (ii)). (b) Serial lipid concentrations generated by LipidXplorer exhibit close agreement with samples prepared via manual pipetting. Data points represent the mean ± SD (*n* = 3 independent experiments); lines show linear regression with corresponding *R* ^2^ values. (c) Ternary system with one lipid channel barcoded aligns with systematic linearity expected with stepwise changes, with a low deviation error (Figure S2d) (d) (i) Representative emission spectra of the Laurdan probe, an indicator of membrane fluidity, measured in particles made using our technology, spanning discrete DOPC:cholesterol compositions (0–60 mol% cholesterol). (ii) Generalized polarization (GP) values extracted from these spectra and compared with results obtained using extrusion and manual microfluidic preparation and showing consistency with the latter. Holm‐adjusted pairwise Welch's *t*‐tests showed no significant difference between LipidXplorer and manual microfluidics (adjusted *p* = 0.370).

We validated LipidXplorer's ability to achieve precise compositional control at high formulation speeds (∼200× faster: 15 s/sample LipidXplorer, 30–60 min/sample extrusion) using its flow‐rate controlled lipid selection strategy. Accurate formulation requires not only precise relative flow rates of individual lipid stocks, but also complete mixing prior to the particle‐formation junction; in lipid systems, incomplete upstream homogenization can alter the downstream self‐assembly pathway and produce polydisperse, aggregated or compositionally heterogeneous particles. To address this, we first assessed mixing performance in custom‐designed advective channels (Figure 2a), designed to work across total lipid flow rates ranging from 3 µL min^−^^1^ to 100 µL min^−^^1^, with the upper limit visualized in Figure 2ai,ii. Because effective lipid particle assembly occurs only within a defined window of total flow rate and aqueous flow‐rate ratio, the upstream advective pre‐mixing geometry was designed to homogenize lipid stocks across the corresponding lipid‐flow‐rate range. In all cases, the three distinct input streams were fully homogenized before particle formation, ensuring that individual particle compositions accurately reflected the intended bulk formulation. This homogeneity enables reliable population‐level compositional analysis using bulk measurement techniques.

Particles synthesized by LipidXplorer—using calibrated flow sensors for concentration determination and automated dispensing into well plates (Figure S2a)—exhibited high compositional fidelity relative to controls prepared via manual pipetting when assessed using fluorescently labeled lipids. In a two‐component system of labeled and unlabeled 1,2‐dioleoyl‐sn‐glycero‐3‐phosphocholine (DOPC) (Figure 2b), the mean deviation from the manually prepared reference was 1.36% across triplicate samples, with both exhibiting high characteristic linearity with *R* ^2^ values over 0.98. Fine compositional tuning was also achieved, with increments as small as 5% (corresponding to 21 discrete flow steps) reproduced with high linearity (*R* ^2^ = 0.9983) and a maximum deviation of 1.99% across 20 compositions measured in three independent runs (Figure S2b). This performance reflects both the robustness of the flow‐control algorithm in maintaining target flow rates (Figure S2c) and the reliability of the automated dispensing process.

Comparable accuracy was observed in a ternary lipid system, in which only one component was fluorescently labeled to avoid Förster resonance energy transfer effects (Figure 2c). In a single‐pass screen without averaged readings (formulated in an order toward Lipid 3 vertex), the measured compositions closely followed the expected linear trends (*R* ^2^ = 0.9997), with a mean deviation of 1.1% and a maximum deviation of 4.4% (Figure S2d). Compared with an identical ternary composition space prepared by manual pipetting (Figure S2dii, iii), agreement between the two preparation methods was high, with a mean compositional consistency of 97.7 ± 1.62% S.D. Together, these results demonstrate that LipidXplorer can reliably generate specified lipid compositions with high accuracy and repeatability, without evidence of systematic bias across the tested compositional space.

The selected concentration range also influences the concentration uncertainty associated with flow control. For a given screening protocol, the same flow‐rate steps are used regardless of stock concentration, but the concentration interval spanned by those steps depends on the difference between the input stocks. Thus, when interpolating between 0 mM and 100 mM stocks, a 2% relative flow‐control error corresponds to a flow‐control‐derived uncertainty of approximately 2 mM. In contrast, using the same flow‐rate ratios to interpolate between more similar stocks, such as 24 and 25 mM, maps the same relative flow‐control error onto a much smaller absolute concentration uncertainty of approximately 0.02 mM (Figure S2e). This scaling applies only to the flow‐control contribution and does not imply an equivalent reduction in total experimental error, as other sources of uncertainty may include absolute components that do not scale with the concentration window. Nevertheless, this principle can be exploited in hierarchical or adaptive workflows, where broad surveys are followed by targeted refinement using more closely matched input stocks around regions of interest.

Beyond compositional accuracy, formulation throughput and reagent consumption are critical for large screening campaigns. Both are primarily determined by the user‐defined total flow rate and collection volume but are also influenced by composition switching time and system dead volume. Owing to the continuous‐flow architecture, sample volumes as low as 20 µL can be collected; however, each formulation incurs reagent loss during flow switching and flushing.

Switching times were consistently <2.5 s and largely independent of total flow rate (Figure S2f), corresponding to volumetric losses of <2.1 µL at 50 µL min^−^^1^ and 41.6 µL at 1 mL min^−^^1^, for example. In addition, a ∼4 µL dead volume between the lipid mixing junction and outlet was flushed three times per composition to minimize cross‐contamination, resulting in a fixed loss of ∼12 µL per sample, in addition to total flow rate‐dependent losses (Equation S3). Although this overhead became more pronounced at high total flow rates—particularly for mRNA‐LNP formulations, due to high mRNA cost—overall reagent consumption remains below that of comparable commercial platforms such as Sunscreen [28] (105 µL/sample). Formulation time per sample was similarly total flow rate‐dependent: collecting 100 µL at 880 µL min^−^^1^ required <15 s per composition, substantially outperforming semi‐automated and manual methods, with longer collection times at lower total flow rates (Equation S4).

#### 2.2.2 Biophysical Screening

To validate the ability of the LipidXplorer platform to screen biophysical phase space, we examined a two‐component DOPC–cholesterol lipid system using the environment‐sensitive membrane probe Laurdan, whose well‐characterized response to bilayer packing and hydration reports the cholesterol‐dependent increase in molecular order and reduction in interfacial water penetration. Since Laurdan's emission is responsive to the polarity of its environment, this reduces the energy loss to dipole–dipole interactions with water, inducing a blue shift in the emission spectra. This shift between the hydrated and dehydrated states can be quantified by using the generalized polarization (GP) metric (Equation S5).

DOPC liposomes containing 0–60 mol% cholesterol were synthesized using the LipidXplorer platform, with dynamic light scattering (DLS) showing a mean hydrodynamic diameter of 148.8 ± 31.5 nm (Figure S2g) and polydispersity 0.068 ± 0.036. Cholesterol content was limited to a maximum of 60 mol% to remain below its reported solubility limit in phospholipid bilayers, beyond which cholesterol‐rich crystalline phases can form [35]. The resulting Laurdan emission spectra for all 12 compositions produced by the platform are shown in Figure 2di. Correlating with the Laurdan‐hydration relationship, a progressive exchange from the 490 nm to 435 nm band was observed with increasing concentrations of cholesterol. Although multiple studies have applied Laurdan GP to DOPC‐cholesterol mixtures [36, 37], reported absolute GP values often vary markedly between laboratories, complicating direct comparison. To verify the compositional accuracy and biophysical fidelity of particles produced by LipidXplorer, several representative formulations were independently prepared using standard 100 nm extrusion and a manual microfluidic method. In each case ethanol was added in the same concentration expected from MHF to remove its polarity difference as a consideration; however, we observed this to have minimal impact on GP at a volume percentage under 11% (Figure S2h). While the overall trends from both comparison methods agreed with the automated measurements, samples produced by extrusion exhibited a consistent negative offset. We attribute this shift to structural differences in vesicle architecture: extruded vesicles frequently contain multilamellar or partially stacked structures [38], which may alter Laurdan's exposure to the aqueous interface and subsequently affect the emission profile. Similar trends were also observed in the 1,2‐dipalmitoyl‐sn‐glycero‐3‐phosphocholine (DPPC)‐cholesterol system (Figure S2i). Importantly, GP shifts demonstrated by manual microfluidic formulation matched LipidXplorer formulation which validates the platform's ability to generate compositionally accurate and biophysically representative vesicle suspensions.

### 2.3 High‐Resolution Biophysical Mapping of Composition Space

**FIGURE 3:**
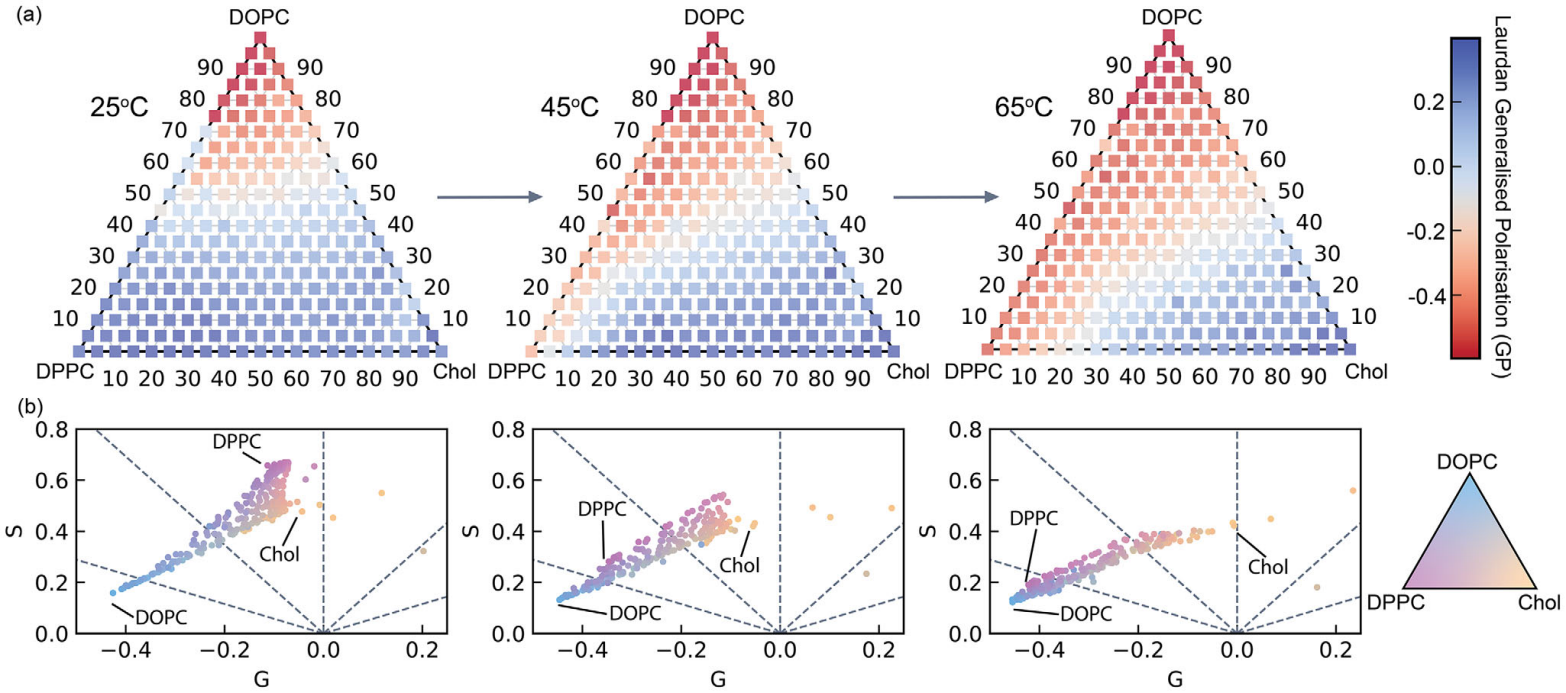
High‐resolution biophysical mapping of composition space. Generation of three‐dimensional biophysical phase space of the DOPC‐DPPC‐cholesterol (Chol) system with the membrane probe Laurdan (a) Ternary composition space mapped with generalized polarization of Laurdan emission spectrum, measured at three temperatures (25°C, 45°C, and 65°C). Data points represent the mean value of *n* = 2 independent screen replicates (b) The same composition space mapped as a spectral phasor with particle composition highlighted by color—composition correlates with spatial position of membrane properties. High‐cholesterol compositions expected to form crystalline structures are observed spatially separated from bilayer‐forming compositions.

To further demonstrate the utility of high‐throughput generation of libraries of lipid compositions using LipidXplorer, we aimed to explore a ternary lipid space. A library of the DOPC‐DPPC‐cholesterol system was produced at unprecedented resolution with increments of 2.5 mol% from 0 to 100—a total of 231 compositions. Samples were synthesized and dispensed over three well plates, with a total synthesis time of 54.7 min. For comparison, to produce the same particles by extrusion, as typical in membrane biophysics, a time of ∼200 h could be expected or ∼14.5 h for the current state‐of‐the‐art microfluidic approach [28]. We also collected only 100 µL per sample—at least a fivefold reduction compared with extrusion—while avoiding the labor‐intensive preparatory steps it requires, including freeze–thaw cycling and lipid hydration into the aqueous phase. Again, Laurdan GP was used as a biophysical probe of each sample and visualized on the compositional phase diagram seen in Figure 3a. Qualitatively, our results reflected established trends [39], pure DOPC exhibited the lowest membrane order and therefore the lowest GP at 25°C, while increasing fractions of DPPC or cholesterol produced progressively higher GP values due to reduced interfacial hydration and increased lipid packing. In mixtures of DOPC and DPPC, phase separation was expected [40], and the Laurdan signal reflected an intensity‐weighted average of the coexisting Ld (liquid‐disordered) and Lβ (gel‐phase) environments, giving intermediate GP values. As temperature increased, the largest GP shifts occurred along the DPPC‐rich edge, corresponding to the melting of DPPC from the Lβ gel phase into a fluid state, while DOPC‐rich compositions remained comparatively unchanged because they were already fluid at all temperatures studied. However, despite capturing these broad trends, GP alone was insufficient to resolve the more complex phase behavior expected across the ternary space. Because the GP metric condenses the full emission spectrum into a single polarity‐dependent scalar, it cannot distinguish between microenvironments that yield similar hydration levels. This leads to GP degeneracy, such as DPPC‐rich compositions and cholesterol‐rich compositions exhibiting nearly identical GP values, even though DPPC‐rich samples reside deep in the Lβ phase whereas cholesterol‐rich samples form Lo (liquid‐ordered) phases with fundamentally different molecular organization.

To gain greater insight from the Laurdan measurements, we applied Fourier transforms to the spectra, with the G and S quantities (defined in Equations 1 and 2) of the transform plotted on Cartesian axes to produce spectral phasors [41]. Figure 3b shows the phasor (cropped to focus on the relevant region) with the composition mapped. In contrast to GP, phasor analysis revealed a strong spatial correlation against composition, with DOPC, DPPC, and cholesterol located at the left, top, and right vertices of the triangle, respectively, and the distribution of mixed compositions between. This improved differentiation was achieved because the phasor considers the molecular changes that broaden, skew, and heterogenize the emission spectrum in ways that GP does not detect. For example, cholesterol‐rich environments are more heterogeneous than DPPC [42], resulting in a broader spectrum. Linear trends are seen along the edges of the triangle for the DOPC‐DPPC and DOPC‐cholesterol systems correlating with incremental changes between two segregated phases [43, 44, 45]. With increasing temperature, this distribution shifted to the left, corresponding to a significant proportion of the DPPC rich particles assuming the fluid phase originally unique to DOPC. This aligns with the standard DPPC transition from the gel to liquid phase as temperature increases. Comparatively, DOPC‐rich compositions remained largely stationary in the phasor with the small changes observed likely a result of the increased molecular energy or the influence of DPPC additions. Several cholesterol‐rich phases lay notably far outside of the triangular bounds. This can be explained by the formation of pure cholesterol non‐lamellar crystalline domains or phases which have radically different morphologies from those of the other particles [46], which we identify beginning from [15, 0, 85]. This creates a vastly different environment for the Laurdan probe, resulting in outlying emission behavior.

These results, which survey the DOPC‐DPPC‐cholesterol composition space at higher resolution than achieved before, are consistent with well‐established historical understanding. However, the limitations of Laurdan spectral analysis prevent distinct resolution of phase behavior. Biophysical characterization could be extended using Laurdan lifetime measurements to resolve coexisting phases and define phase boundaries more precisely. In the future, the full potential of such libraries will be realized by integrating them with plate‐based analyses, including high‐throughput DLS–Zeta, small‐angle/wide‐angle x‐ray scattering [47], and nano‐flow cytometry. Residual ethanol, while a concern for membrane biophysics, can be addressed using plate‐based dialysis and buffer exchange. Altogether, this large‐scale screening strongly supports the further use of the LipidXplorer platform to survey areas for characterizing phase‐space environments. The ability to produce large libraries of biophysically distinct particles within such a short timeframe may provide an advantage to soft nanoparticle research which should not be underestimated.

### 2.4 Benchmarking Clinically Approved mRNA‐lipid Nanoparticles

**FIGURE 4:**
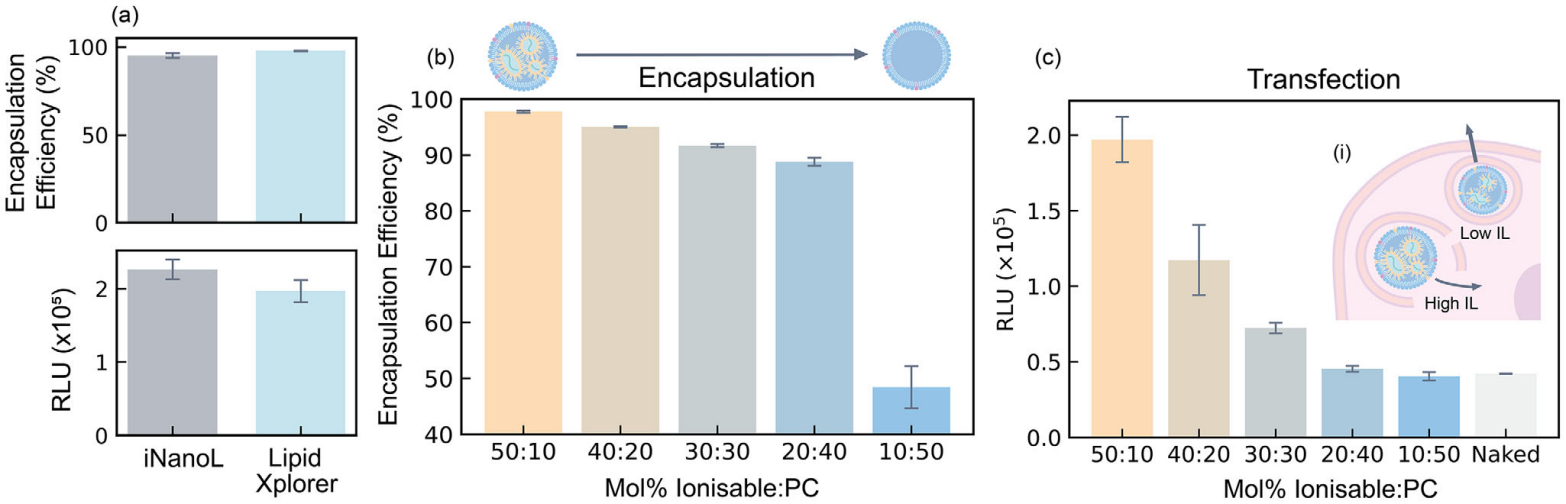
Screening of mRNA‐loaded lipid nanoparticles. All data are presented as mean values ± SD (*n* = 3) (a) Comparison of encapsulation and transfection efficiencies between the SM102:DSPC:cholesterol:DMPE‐PEG2000 systems (50:10:38.5:1.5) prepared using LipidXplorer and a commercial lipid nanoparticle formulation device (INanoL+). Luciferase expressing mRNA was used and corresponding luminescence in relative light units (RLU) was measured. (b) The ionizable lipid SM102 was exchanged for DSPC in the automated formulation screen. Encapsulation of luciferase mRNA decreased predictably with reducing attractive interactions between the lipids and mRNA. (c) Variations in transfection efficiency in response to the SM102‐DSPC composition.

Given the key role of mRNA delivery for applied lipid nanotechnologies [1], we demonstrate that the platform can formulate and screen clinically approved lipid nanoparticles (LNPs) across stepwise compositions, achieving boosted encapsulation and comparable transfectability to a commercial platform. For effective LNP synthesis, the MHF geometry was replaced with an advective mixing device (Figure S3a), which is better suited to achieve complete mixing of the lipid and aqueous phases, leading to higher encapsulation and small particles (Figure S3b). The SM102–DSPC–cholesterol–DMPE‐PEG2000 lipid system was selected as a representative test case, with SM102 serving as the ionizable lipid that enables mRNA encapsulation and promotes endosomal escape following cellular uptake, consistent with its use in the clinically approved mRNA‐LNP vaccine formulation [48]. A standard molar composition of 50:10:38.5:1.5 was manually prepared as a pre‐mixed ethanol stock then formulated using a single input line of LipidXplorer to benchmark against LNPs produced using a commercial formulation system (Figure 4a).

The encapsulation efficiency obtained with LipidXplorer was marginally higher (+2.9%) than that achieved using a commercial LNP generation device (INanoL+). Transfection signals in HEK‐293 cells, while robust, displayed a small (13.1%) reduction relative to those obtained with the commercial formulation system. This difference could be attributed to the reduced total flow rate (480 µL min^−1^) employed by the LipidXplorer platform, which minimizes reagent consumption per condition but increases the characteristic diffusion length during mixing, resulting in the formation of larger particles [49, 50]. The LNPs produced using the LipidXplorer platform exhibited a mean hydrodynamic diameter of 189.5 ± 9.1 nm, compared to ∼100 nm expected for the commercial system. Although this size remains within a range that is acceptable for in vitro delivery and adequate for first‐round screening, increased particle dimensions are expected to affect cellular uptake efficiency or alter endosomal trafficking. For studies in which particle size is a critical parameter, operation at higher total flow rates may therefore be advantageous, but at the expense of waste and cost (Equation S3). We also note that ultrafiltration was used here to enable comparison with standard preparation methods. For truly high‐throughput LNP screening, however, automated approaches for pH adjustment and ethanol removal will be required, and these additional steps may themselves influence particle size.

An automated linear compositional screen was subsequently performed to investigate the effect of the ionizable lipid SM102 content on particle performance (Figure 4b). Two stock solutions for either end of the compositional scale (molar ratios of 0:60:38.5:1.5 and 60:0:38.5:1.5 SM102:DSPC:cholesterol:DMPE‐PEG2000) were manually prepared, input into the LipidXplorer and varied stoichiometrically to modulate SM102 content while maintaining constant cholesterol and DMPE‐PEG. The encapsulation efficiency decreased smoothly as the molar fraction of the ionizable lipid was reduced from 50 mol% to 20 mol%, with a more pronounced decline observed at 10 mol%. This trend is consistent with reduced electrostatic interactions between positively charged SM102 and mRNA [51] under acidic formulation conditions, which diminish the driving force for complexation. At 10 mol% SM102, the nitrogen‐to‐phosphate (N/P) ratio approaches unity, resulting in the loss of excess cationic charge and increased susceptibility to incomplete complexation [52], consistent with the reduced encapsulation efficiency of 40.3 ± 3.2%. For studies where the N/P ratio must remain constant, an additional buffer line may be added to control the mRNA concentration at the point of formulation.

The LNPs generated across this compositional series were subsequently dosed to HEK‐293 cells with equal moles of mRNA, with transfection efficiency exhibiting a positive correlation with the SM102 content (Figure 4c). While this behavior partly reflected the reduced mRNA encapsulation, it also arose from differences in intracellular delivery efficiency. Ionizable lipids facilitate endosomal escape through charge‐mediated membrane destabilization and the formation of fusogenic non‐lamellar phases [53, 54]. Delivery remained strongly dependent on SM102 content, indicating that reduced functional output cannot be explained by encapsulation efficiency alone [55]. For example, the 20 mol% SM102 formulation exhibited transfection levels comparable to those observed for naked mRNA controls, despite an encapsulation efficiency of 88.8 ± 0.5%. Given HEK‐293 readily takes up particles, we attribute this difference to role of SM102 in enabling endosomal fusion and escape. This behavior defines a functional lower bound for ionizable lipid content (20 mol%) within LNPs of this type.

These results demonstrate the capability of LipidXplorer to reproduce the established LNP composition–function relationships while enabling low‐volume, automated formulation and screening, comparable to existing LNP‐focused technologies [28, 29]. The scope of the present study was constrained by the cost of mRNA and the time required for cell‐based assays, highlighting a trade‐off between biological validation depth and material‐efficient screening. Platforms capable of formulating single compositions at sub‐10 µL volumes, together with the development of reliable cell‐mimetic screening models, could substantially expand access to systematic nanoparticle screening within academic research constraints.

### 2.5 High‐Throughput Screening for Functional Discovery

A central goal of screening methodologies is the identification of novel and optimal material compositions for a given functional task. Achieving this typically requires a combination of large‐scale screening to establish global composition–function relationships, followed by local optimization through either rational selection or AI‐driven exploration [56]. Here, we apply the LipidXplorer platform to interrogate the functional composition space of fusogenic non‐lamellar particles [57]. These particles hold promise as next‐generation delivery vehicles [58], as they can bypass uptake and endocytic barriers through direct fusion with the cell membrane, thereby avoiding complex and often unpredictable internalization pathways that vary with particle morphology, cell type, and cargo. In parallel, fusogenicity is advantageous for synthetic biology, enabling reliable, charge‐independent transport into synthetic cells for both aqueous reagents [59] and membrane‐associated proteins [60]. Rational engineering of such particles for both applications requires systematic screening, characterization, and optimization of their fundamental biophysical behavior in well‐defined model environments.

**FIGURE 5:**
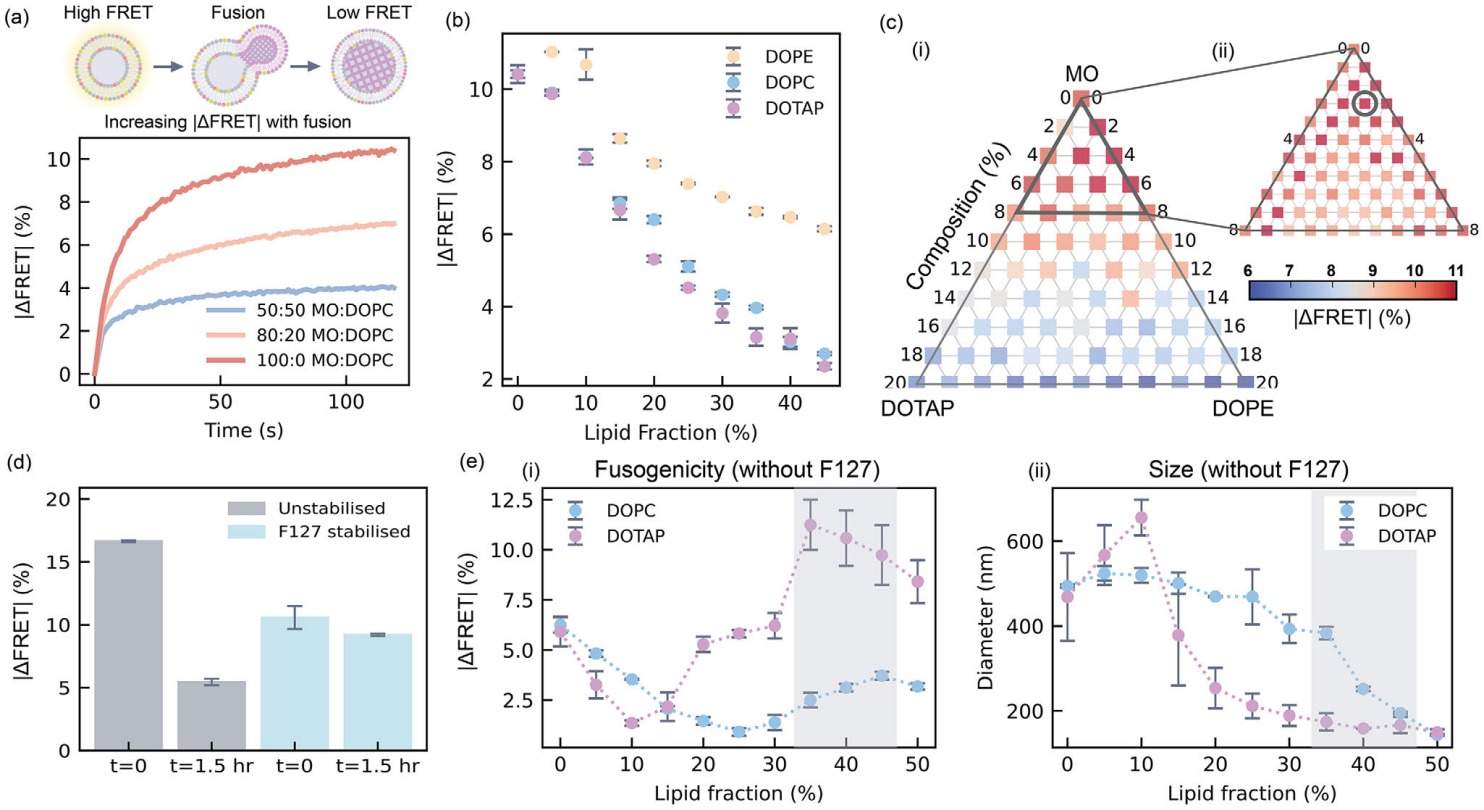
High‐throughput screening for discovery of fusogenic non‐lamellar nanoparticles. (a) Schematic of the FRET‐based fusion assay. Fusion between fluorescently labeled substrate vesicles and Monoolein (MO)‐based particles dilutes donor–acceptor pairs, resulting in reduced FRET efficiency. The magnitude of the difference between the initial and final state |ΔFRET| is visualized over time for three representative compositions extracted from the data in (b). Kinetic curves show a single representative trace (*n* = 1). (b) Fusogenicity trends for MO particles doped with lamellar‐forming lipids. Increasing DOPC/DOTAP content produces a monotonic decrease in fusion, whereas low fractions of DOPE induce a modest enhancement. Mean values ± SD (*n* = 3). (c) Compositional screening of the ternary MO–DOTAP–DOPE system. (i) Fusogenicity maxima are observed at low molar fractions of DOTAP and DOPE, motivating higher‐resolution exploration of this regime (ii) to identify critical compositions. (d) Fusogenicity of MO particles with and without a 10 mol% Pluronic F‐127 stabilizer measured at *t* = 0 h and *t* = 1.5 h. Mean values ± SD (*n* = 2). (e)(i) Optimization of fusogenicity in the absence of stabilizing polymer, measured 1.5 h post‐formulation. Addition of lamellar‐forming lipids stabilizes particle membranes and yields a fusogenic maximum at approximately 35–45 mol% lipid content. Mean values ± SD (*n* = 2) (ii) Accompanying size characterization for the unstabilized system MO‐based particles, revealing decreased hydrodynamic diameters with significant addition of the lamellar lipids. Mean values ± SD (*n* = 2).

To quantify fusogenicity, we applied a Förster Resonance Energy Transfer (FRET) assay [61, 62]. Non‐lamellar (unlabeled) particles are incubated with DOPC vesicles containing 1% 18:1 NBD‐PE and 1% 18:1 Lissamine rhodamine‐PE which form FRET pairs. Fusion leads to membrane dilution and spatial separation of the fluorophores, producing a decrease in FRET efficiency; the rate and magnitude of this change report on the fusogenic behavior of the particle population (Figures 5a and S4a). More fusogenic compositions produce faster and larger FRET changes, reflecting an increased frequency and extent of lipid mixing. We found that the progressive addition of the lamellar‐forming lipid DOPC to monoolein (MO) particles (stabilized with 1 mol% polymer F‐127 and previously shown to directly form cubosomes with an identical MHF synthesis method [59]) reduced fusogenicity, yielding increasingly shallow kinetic profiles. A two‐component screen spanning 0–50 mol% DOPC in MO revealed an approximately linear decrease in fusogenicity, with near‐complete loss at 50 mol%, coincident with the transition to a lamellar membrane phase (Figure 5b). Interestingly, incorporation of DOPE produced a local maximum in fusogenicity at ∼5 mol%, followed by a gradual decline extending beyond 50 mol%. This behavior is consistent with DOPE's preference for highly negative curvature, which lowers the energetic barrier to membrane fusion by stabilizing stalk intermediates [63].

We next expanded the compositional space to include the cationic lipid DOTAP, which is expected to interact electrostatically with the negatively charged phosphate head groups of DOPC [64], and performed a ternary screen with up to 20 mol% total additions to MO. Broadening the compositional space enabled access to improved formulations but introduced multiple interacting physical effects that challenge rational prediction. A coarse survey (Figure 5ci) using 2 mol% compositional increments identified the most fusogenic particles at low additive concentrations (∼0–6 mol%). At these levels, DOPE introduced curvature stress [65] while DOTAP contributed favorable electrostatic interactions; beyond this regime, both components reduced fusogenicity, likely forming more stable membrane structures [64].

Having identified this critical region of phase space, we performed a second, higher‐resolution screen using 0.8 mol% increments (Figure 5cii). This was achieved by adjusting lipid stock concentrations, enabling increased compositional precision while maintaining experimental variability dominated by flow parameters rather than formulation error. The most potent composition was identified at 97.6:0.8:1.6 mol% MO:DOTAP:DOPE, yielding a maximal FRET change of 11.1%, but only marginally higher than neighboring compositions (0.123% standard deviation between nearest neighbors). Together, these results indicate that fusogenicity emerges from a finely balanced interplay between curvature stress and electrostatic interactions, giving rise to a narrow compositional optimum. The shallow landscape surrounding the maximum underscores the challenge of identifying functional extrema and highlights the value of combining broad screening with targeted local refinement to resolve subtle energetic effects.

Having located an optimal composition, we next focused on the relationship between stability and fusogenicity that governs the function of non‐lamellar particles. When synthesized in the presence of Pluronic F‐127, the polymer adsorbs at the lipid–water interface, stabilizing the high interfacial curvature associated with the non‐lamellar phase [66]. Although this enhances long‐term colloidal stability [67], the polymer corona introduces a steric barrier that increases the entropic cost of membrane fusion (Figure S4b) [59, 66]. We observe that the addition of Pluronic F‐127 diminishes fusogenic interactions with target membranes, evidenced by a reduced FRET response compared to unstabilized particles when mixed in the instant after formulation *t* ≈ 0 h (Figure 5d). Over time, however, stabilized particles retain their fusogenicity, whereas unstabilized particles become significantly less fusogenic as seen at *t* = 1.5 h. This decline occurs rapidly, with a 36.1% drop within the first 10 min post‐formulation (Figure S4c). DLS also reveals, in the absence of polymer stabilization, that the particles exhibit a greatly increased particle size of 495.2 ± 13.1 nm, compared to 131.6 ± 14.6 nm when stabilized with F‐127. We observe this from our earliest timepoint *t* = 5 min but surprisingly sizes of both stabilized and unstabilized remain consistent over the 30 min measurement period (Figure S4d). Given it is well characterized that small cubosomal particles exhibit high curvature and high fusogenicity [59], particle size is likely to be a key factor in these observed fusogenic behavior differences. Coarsening of unstabilized particles in the moments after formulation is consistent with previous reports [68], while the observation of decreasing fusogenicity despite stable size over 5–30 min may suggest reconfiguration toward lower energy states. We aim to investigate this further through time‐resolved x‐ray diffraction studies to characterize structural evolution after formulation.

To achieve a balance between stability and fusogenic potency without relying on polymeric stabilizers, we explored lipid composition as a stabilization strategy. Particles incorporating varying amounts of DOPC or DOTAP into MO were synthesized in phosphate‐buffered saline (PBS) and incubated for 1.5 h at room temperature prior to fusogenicity assessment. The resulting trends (Figure 5ei) differed markedly from those observed in the F‐127‐stabilized systems. At low molar fractions, both DOPC and DOTAP reduced fusogenicity, with ΔFRET decreasing to ∼1.5%. We attribute this behavior to the incorporation of cylindrical lipids into the monoolein‐rich membrane, where they disrupt the native inverse‐cone packing of monoolein and further destabilize the particles. This is supported by the increased particle size observed relative to pure MO (Figure 5eii), which suggests membrane destabilization and coarsening has occurred—ultimately leading to reduced fusogenicity. Strikingly, at higher lipid contents we observed a recovery, and subsequent enhancement, of fusogenicity at the 1.5 h time point, emerging at ∼30 mol% for both DOTAP and DOPC and peaking shortly thereafter. This fusogenic character may be partly explained by particle size. At high levels of cylindrical lipid doping, the particles reached diameters below 150 nm, a threshold previously associated with an increase fusogenicity [59]. Interestingly, while size remains low up to 50 mol% additions, the fusogenicity begins to decrease, suggesting a compromise between colloidal stability and maintaining non‐lamellar fusogenic phases. This character is something we intend to validate structurally in future work. We speculate this behavior may reflect approaching a compositionally driven phase boundary between cubic and lamellar regimes [68], where at this boundary, partial phase coexistence or segregation stabilizes the particle architecture while preserving local domains of high curvature. Such interfacial or heterogeneous structures may suppress self‐fusion and coarsening yet retain the capacity to form fusion stalks upon contact with a purely lamellar membrane, thereby restoring fusogenic activity. Unlike the unstabilized formulations, increasing lipid fraction in the stabilized system did not restore fusogenicity. This suggests that fusogenicity is not determined solely by total lipid content, but critically by interfacial organization. Polymer‐mediated stabilization may introduce a steric or kinetic barrier that limits membrane contact and/or alters surface arrangement [69], preventing fusion at higher cylindrical lipid doping.

## 3 Conclusion

We present LipidXplorer as a versatile and scalable platform for rapidly formulating compositionally defined lipid particles across multiple architectures. By integrating continuous‐flow microfluidics with robotic collection, the system delivered accurate, highly reproducible formulations at ∼14 s per composition—over 200 times faster than extrusion—while maintaining reagent consumption comparable to commercial LNP instruments. LipidXplorer's contribution lies in the strategic adaptation of well‐established microfluidic aqueous‐screening principles and robotics to the specific formulation, compositional and collection requirements of lipid particles, while retaining broad accessibility and compatibility with alternative microfluidic devices and laboratory workflows.

Using this platform, we mapped the DOPC–DPPC–cholesterol phase space at unprecedented resolution, recovering canonical compositional and thermal trends and demonstrating that high‐density biophysical mapping—previously impractical due to labor and time—is now readily achievable. LipidXplorer also formulated mRNA‐loaded LNPs, reproducing expected dependencies of encapsulation and transfection on ionizable lipid content and validating applicability to clinically relevant nanoparticle classes. Finally, we exploited the platform to investigate the functional fusogenicity of a comparatively underexplored phase space by doping monoolein‐based particles with additional lipid components. High‐resolution screening of the MO–DOPE–DOTAP system revealed distinct compositional optima, and subsequent experiments highlight the potential of lipid‐based strategies as an alternative to polymeric stabilization. Notably, we identified a previously unreported local maximum near 40 mol% DOTAP/DOPC, suggesting a stabilizing contribution from the cubic–lamellar phase boundary.

Together, these results establish LipidXplorer as an accessible, modular and automation‐ready technology for navigating complex particle phase landscapes. Its compatibility with high‐throughput workflows and machine learning pipelines provides a foundation for data‐driven discovery, enabling researchers to uncover structure–function relationships that remain opaque to conventional formulation methods.

## 4 Methods

### 4.1 Manufacture of Microfluidic Chip

Microfluidic geometries (Figures 2a and S3) were designed in AutoCAD (Autodesk, USA), derived from Pilkington et al. [59], and imported into Inventor (Autodesk, USA) to generate three‐dimensional models, with channel heights extruded to 200 µm. The models were subsequently imported into DeScribe (NanoScribe) for slicing and compilation prior to printing using a NanoScribe platform (NanoScribe, Germany). Silicon wafer substrates (Inseto, UK) were sequentially washed, plasma treated and functionalized by incubation in 5% (v/v) A174 silane (Merck, Germany) with an acetic acid catalyst for 30 min.

Microfluidic molds were printed in IP‐S photoresist (NanoScribe, Germany) directly onto the prepared substrates using a 21× immersion objective. Printing parameters were as follows: laser power, 70%; shell scan speed, 100 000; scaffold scan speed, 150 000; base scan speed, 7500. Following printing, wafers were immersed in propylene glycol monomethyl ether acetate (PGMEA; Merck) to remove uncured resin for 30 min, rinsed five times with isopropanol, and dried under a nitrogen stream.

Polydimethylsiloxane (PDMS; Dowsil Sylgard 184) was prepared at a 10:1 base‐to‐curing‐agent ratio, degassed for 1 h, poured over the molds, and cured at 70°C for 1 h. Devices were cut from the molds, and inlets were formed using a 0.75 mm biopsy punch (Rapid‐Core, Darwin Microfluidics). Both components were washed with ethanol, dried with compressed air, and cleaned using adhesive tape (Scotch Magic Tape), followed by oxygen plasma treatment (Diener Zepto, Diener Electronic, Germany) for 20 s. Bonding was completed by bringing the treated surfaces into contact and baking at 70°C for 30 s. Final devices were inspected for contamination, cracks, and channel occlusions prior to use. Devices were only used for a single experiment.

### 4.2 Pressure‐Driven Microfluidic Flow

Four independent pressure‐driven channels were connected to a computer‐controlled pressure regulator (OB1 Mk3 0–2 bar, Elveflow, France), driving either 1.5 mL lipid reservoirs or a 50 mL buffer reservoir. Pressure control was implemented using custom Python code interfacing with the regulator via the Elveflow software development kit (SDK). The control code is publicly available on GitHub (https://github.com/bdigg/lipidxplorer; “Pump.py”).

Flow rates in each channel were monitored in real time using inline thermal flow sensors sampling every 0.4 s (LG16‐0431D/0150D, Sensirion, Switzerland). To stabilize flow downstream of each sensor, 4 cm of 65 µm inner‐diameter capillary tubing was installed before connection to the microfluidic device. Tygon ND 100‐80 tubing (0.51 mm ID 1.52 mm OD) was used for all other fluidic connections, with 1/4‐28 flangeless fittings. Blunt stainless steel connectors (23G) were used to interface the tubing with the microfluidic chip inlets. Five centimeter of Tygon ND 100‐80 tubing (0.25 mm ID, 0.76 mm OD) was installed between the chip outlet and a 1‐in. stainless‐steel connector which was positioned to act as an outlet nozzle 2 mm from the top of the plate. A schematic of the microfluidic setup is provided in Supporting File 1. Unless otherwise stated, tubing, biopsy punches, and microfluidic fittings were obtained from Darwin Microfluidics, France.

All microfluidic tubing was replaced after each experiment, with permanent components such as connectors and flow sensors rinsed with 70% ethanol, soaked in Hellmanex II (Merck) for 15 min, before rinsing with deionized water and air‐drying.

### 4.3 Calibration of Flow Sensors

Flow sensors were calibrated using a gravimetric method. Pre‐weighed 1.5 mL tubes were sealed with Parafilm, and individual sensors were cleaned with Hellmanex II before connection to a pressure line and fluid reservoir. After stabilizing flow at a target pressure, outlet tubing was inserted into the tubes, and fluid was collected until approximately 1.5 mL was dispensed. Collected mass, fluid density and collection time were used to calculate the true volumetric flow rate. Calibration was performed across multiple flow rates using water (buffer lines) and ethanol (lipid lines) with three repeats. Calibration curves were generated by fitting measured flow rates to raw sensor outputs using linear or range‐limited polynomial relationships, which were subsequently applied to correct sensor readings during experiments. Calibration accuracy was verified periodically by connection to a syringe pump driving 7 mM 1,2‐dioleoyl‐sn‐glycero‐3‐phosphocholine (DOPC, Avanti) in ultra‐dry ethanol through Hamilton gastight syringes (Hamilton, USA).

### 4.4 Construction of LipidXplorer Platform

Three linear actuators (V‐Slot Mini V Linear Actuator; 0.26 mm positioning accuracy, OpenBuilds Partstore, USA) were assembled in an H‐frame configuration and secured to a rigid base plate via tee‐slot 90° connectors. Each actuator was driven by a NEMA 17 stepper motor (OpenBuilds), with motor windings connected to a TB6600 stepper motor driver (Farnell, UK) at 1/8 micro‐stepping. This results in well‐to‐well travel times of ∼0.6 s, with movement overlapping with the flow switching process. Motor control was implemented using an Arduino Nano Every (RS Components, UK), which communicated with the host computer via serial interface and relayed step and direction signals to the motor driver. Movement commands specified both direction and number of motor steps, corresponding to defined linear displacements along each axis.

Limit switches (OpenBuilds) were installed on both axes to enable homing routines and establish a reproducible positional reference for subsequent movements. Alignment to the well plate (placed in a fixed‐position holder) was achieved through a one‐time manual adjustment, positioning the fluid outlet above the center of the first well; the corresponding offset from the home position was incorporated into the automated initialization sequence. Custom housing for microfluidic components was designed in Autodesk Fusion 360 and fabricated in ABS using an Ultimaker 2 3D printer. The flow switch plate was 3D printed in clear biomedical resin (Liqcreate) using an Elegoo Saturn Ultra and actuated using a micro servo (SG92R, RS Components), controlled by the same Arduino system, with a switching time of 1.2 s between the waste and collection states. Arduino code, including details on pin positions, is publicly available on GitHub (https://github.com/bdigg/lipidxplorer; “autoexpel.ino”).

A vacuum pump (SparkFun, RS Components) was connected to the switch‐plate via 40 cm Tygon tubing, extracting to a 40 mL Falcon tube. All code governing robotic positioning and automated sample expulsion is publicly available on GitHub (https://github.com/bdigg/lipidxplorer; “Expel.py”). Images of the assembled LipidXplorer platform are provided in Figure S1.

### 4.5 General Platform Operation

For each formulation, target compositions were converted into lipid‐line flow rates using the LipidXplorer control software. Flow rates were adjusted at a default period of 0.5 s by the PI controller until all channels reached the 2 µL min^−1^ tolerance, after which the outlet was diverted to collection into the target well. Unless otherwise stated, each formulation was collected sequentially at 100 µL with ∼10.2 µL dead‐volume flush between compositions.

### 4.6 Compositional Validation by Fluorescent Barcoding

Seven millimolar solutions of 1,2‐dioleoyl‐sn‐glycero‐3‐phosphocholine (DOPC, Avanti) in absolute ethanol were doped with 1 mol% 18:1 NBD‐PE or 18:1 Lissamine Rhodamine‐PE (Avanti) or left unlabeled. Mixing performance was assessed using fluorescence imaging at the entrance and exit of the mixing channels on an inverted fluorescence microscope (Nikon TS2). Acquired images were analyzed in Fiji to quantify fluorescence intensity profiles across the channel width and verify complete homogenization.

Barcoding experiments were performed with equal lipid concentrations as above and formulated with PBS at a total flow rate of 480 µL min^−1^ and flow rate ratio of 1:10 using the LipidXplorer platform. Flow rate intervals were set to regular steps and calculated via automated control code (https://github.com/bdigg/lipidxplorer; “flow_calculator.py”), maintaining a constant total flow rate and ratio. Bulk particle fluorescence intensity was measured using a microplate reader (CLARIOstar, BMG Labtech) following extraction of equal sample volumes from each well. A fixed gain of 1500 was applied for all measurements.

To validate composition, manually prepared formulations were generated by combining lipid stocks in chloroform at the desired molar ratios. The solvent was evaporated to form dry lipid films, which were then rehydrated in dry ethanol to 7 mM and introduced by rapidly pipetting into the bulk aqueous phase. This technique is expected to form polydisperse particles; however, the bulk compositions will remain accurate to the stock input. The aqueous‐to‐lipid volume ratio was matched to the flow‐rate ratio used for the corresponding LipidXplorer output. Fluorescence measurements were then performed as described above.

### 4.7 Membrane Biophysical Studies Via Laurdan Probe

Lipid solutions of DOPC, 1,2‐dipalmitoyl‐sn‐glycero‐3‐phosphocholine (DPPC) and cholesterol (Avanti) were prepared at either 5 mM or 10 mM, depending on the experimental composition range. Lipid stocks in chloroform were combined as required, and the solvent was evaporated under a stream of nitrogen to form a thin lipid film. Films were further dried under vacuum in a desiccator for 30 min before resuspension in absolute ethanol containing Laurdan at a 1:200 molar ratio (25 µM Laurdan for 5 mM total lipid). The suspension was gently heated and vortexed until the lipid film was fully dissolved. Lipid–Laurdan stocks in ethanol were stored at −20°C, protected from light, until further use.

For automated formulation, 100 µL liposome dispersions were generated and collected into 96‐well plates using the LipidXplorer platform at a flow‐rate ratio of 10 (80 µL min^−^^1^ lipid stream, 800 µL min^−^^1^ PBS buffer; pH 7.64), with lipid flow rates determined by the algorithm. Fluorescence emission spectra were acquired using a microplate reader (CLARIOstar, BMG Labtech) with excitation at 350 nm. For temperature‐dependent measurements, samples were equilibrated at 5°C increments with a 5 min incubation period prior to acquisition. For large phase‐diagram measurements, an average of two repeats is presented, excluding outliers.

Manually prepared compositions were generated by mixing lipid stocks in chloroform at the desired ratios followed by solvent evaporation to form lipid films. For manual microfluidic hydrodynamic focusing (MHF), films were dissolved in ethanol containing Laurdan and processed through an MHF chip one‐by‐one with PBS buffer at a flow rate ratio of 10, total flow rate 800 µL min^−^^1^. For extrusion‐based preparations, lipid films were rehydrated in PBS to yield 1 mM lipid suspensions, subjected to seven freeze–thaw cycles and extruded eleven times through a 100 nm polycarbonate membrane using a Mini‐Extruder (Avanti). To match solvent conditions in the MHF device, 9 vol% ethanol was added to samples prior to extrusion.

Generalized Polarization was calculated as the ratio between the 435 nm and 490 nm emission peaks (Equation S5). Laurdan fluorescence spectra were analyzed using the spectral phasor Fourier transform method [43]. The real and imaginary components are calculated as G and S, defined below. 𝐼(λ) is the Laurdan intensity as a function of wavelength for a given composition. The cartesian position correlates with the spectral location (polar angle) and broadness of spectrum (polar radius).

(1) $$G=\frac{\int_{\lambda_{\min}}^{\lambda_{\max}}I\left(\lambda \right)\cos\left(\frac{2\pi \left(\lambda -\lambda_{i}\right)}{\lambda_{\max}-\lambda_{\min}}\right)d\lambda }{\int_{\lambda_{\min}}^{\lambda_{\max}}I\left(\lambda \right)d\lambda }$$

(2) $$S=\frac{\int_{\lambda_{\min}}^{\lambda_{\max}}I\left(\lambda \right)\sin\left(\frac{2\pi \left(\lambda -\lambda_{i}\right)}{\lambda_{\max}-\lambda_{\min}}\right)d\lambda }{\int_{\lambda_{\min}}^{\lambda_{\max}}I\left(\lambda \right)d\lambda }$$

### 4.8 DLS Measurements

MHF samples were diluted 10‐fold in their storage buffer and transferred to a NUNC 384‐well clear‐bottom plate. Hydrodynamic particle diameter was determined by dynamic light scattering at 25°C using a Wyatt Möbius DLS plate reader (Wyatt Technology, USA). For each sample, the reported values correspond to the average of five consecutive measurements.

The polydispersity index (PDI) of the particle size distribution was calculated from the intensity‐weighted size distribution as provided by the instrument software. Mean nanoparticle diameter and its standard deviation are reported based on these measurements.

### 4.9 Plasmid DNA Synthesis and Purification

The plasmid DNA (pDNA) construct encoding for firefly luciferase (fLuc) gene (GenBank: AB762768.1) was transformed into *Escherichia coli* and grown in 100 mL cultures in lysogeny broth (LB) media with 100 µg/mL carbenicillin (Sigma‐Aldrich, USA). Isolation and purification of the pDNA were performed using a Plasmid Plus maxi kit (QIAGEN, UK) and a NanoDrop One Microvolume UV–Vis Spectrophotometer (ThermoFisher, UK) was used to measure the concentration and purity of the pDNA. Prior to RNA in vitro transcription (IVT), pDNA was linearized using MluI‐HF (New England Biolabs, UK) for 3 h at 37°C.

### 4.10 mRNA Synthesis, Purification, and Gel Electrophoresis

mMESSAGE mMACHINE kit (Thermo Fisher Scientific) was used to synthesize capped mRNA encoding fLuc through IVT for 2 h at 37°C. After synthesis via IVT, RNA was purified using lithium chloride (LiCl) precipitation. In brief, RNAs were mixed with equal volume of 7.5 M LiCl (Invitrogen) in a 1.5 mL tube by pipetting up and down and then placed at −20°C for at least 30 min. The RNA/LiCl mixture was then centrifuged at 14 000 rpm for 20 min at 4°C. After centrifugation, all liquid was removed without disturbing the pellet and the pellet was washed with 900 µL of 70% ethanol by pipetting up and down. After washing, the pellet in 70% ethanol was centrifuged at 14 000 rpm for 5 min at 4°C. All the ethanol was removed from the pellet, and the pellet was left to dry with the tube open for 5 min. The pellet was then resuspended with 100 µL RNase‐free water and kept on ice for 5 min to allow time for the RNA pellet to reconstitute. To ensure the pellet was properly dissolved and that the final RNA product in water was clear, the RNA was pipetted up and down. RNA concentrations were measured using the NanoDrop One microvolume UV–Vis Spectrophotometer (Thermo Fisher Scientific).

To assess the quality of the RNA, the RNA Millennium Marker Ladder (ThermoFisher, UK) and the purified RNA were mixed with 2× RNA loading dye (ThermoFisher, UK) and incubated at 50°C for 30 min to denature the RNA. A 1.2% agarose gel with 1× NorthernMax Running Buffer (ThermoFisher, UK) was prepared. After incubation, the denatured ladder and RNA sample were added to the gel and the gel was run at 80 V for 45 min. The gel was then imaged on a GelDoc‐It2 (UVP, UK).

### 4.11 mRNA‐Lipid Nanoparticle Formulation Using INanoL+ Microfluidic Device

The following lipid components were used to formulate mRNA in the LNP: SM102 (Biorbyt, USA), 1,2‐distearoyl‐sn‐glycero‐3‐phosphocholine (DSPC) (Avanti), cholesterol (Merck) and 1,2‐dimyristoyl‐sn‐glycero‐3‐phosphoethanolamine‐*N*‐[methoxy(polyethyleneglycol)‐2000] (DMPE‐PEG2000) (Avanti) at a molar ratio of 50:10:38.5:1.5. All lipid components were dissolved in absolute ethanol at a stock concentration of 20 mg/mL. mRNA solutions were prepared in 50 mM sodium acetate at pH 4.5. The N/P ratio used was 6. RNA‐LNPs were formulated using the INanoL+ (XGen Bio, China), where the flow rate ratio of lipid to RNA was 1:3 and the total flow rate was 20 mL min^−1^. The formulated RNA‐LNPs were diluted in 1× Dulbecco's phosphate‐buffered saline with 10% sucrose (w/v) five times the volume of formulated material and was then concentrated through a VivaSpin 6 filter tube (Sartorius, Germany) that has a 10 kDa molecular weight cut‐off (MWCO) at 4000 rpm, 18°C until the desired concentration is achieved.

### 4.12 mRNA‐Lipid Nanoparticle Formulation Using LipidXplorer Platform

For the single test composition, 50:10:38.5:1.5 SM102:DSPC:cholesterol:DMPE‐PEG2000 was prepared as described for the INanoL+ platform. The lipid mixture was then formulated into LNPs using the LipidXplorer platform with only a single lipid inlet line active (the remaining two were blocked). Formulation was performed using an advective‐mixing microfluidic geometry (Figure S3a) at a flow‐rate ratio of 3:1 aqueous:ethanol and a total flow rate of 480 µL min^−^^1^, using the same mRNA buffer solution as the INanoL+ control. All other operational parameters were kept consistent with standard LipidXplorer screening runs where lipid compositional mixing is active.

For the linear screen, lipid stocks were prepared in absolute ethanol as two end‐point solutions (molar ratios of 0:60:38.5:1.5 and 60:0:38.5:1.5, at 14 mM each to maintain stoichiometric exchange of lipids when modulating flow rates). These were loaded onto two of the input lines of the LipidXplorer as the stock solutions which were systematically varied to produce multiple compositions. Fixed flow rate steps were defined to exchange the two input solutions between 0 µL min^−1^ to 120 µL min^−1^, maintaining a constant total lipid flow rate. The formulated particles were post‐processed as described above before transfection.

### 4.13 Quantification of Encapsulation Efficiency and Final RNA Concentration in LNP

Quant‐iT RiboGreen assay (Thermo Fisher Scientific) was used according to the manufacturer's instructions to quantify the encapsulation efficiency and the amount of mRNA encapsulated inside the LNP. In brief, RNA‐LNP samples were diluted in 1× tris‐EDTA (TE) buffer to 6 µg/mL and were then mixed with either 1× TE buffer or 2% Triton X‐100 at a 1:1 ratio in a black 96‐well Costar plate (Thermo Fisher Scientific). RNA standards were prepared according to the manufacturer's instructions and pipetted into the same 96‐well Costar plate. Both samples and standards were incubated at 37°C for 10 min. RiboGreen reagent was diluted in 1× TE buffer at a 1:100 dilution and 100 µL of the diluted RiboGreen reagent was added into each well. The plate was analyzed on the GloMax Microplate Reader (Promega) at an excitation of 480 nm and emission of 520 nm. Based on the standard curve, the concentration of total mRNA and non‐encapsulated mRNA were calculated. The concentration of encapsulated mRNA was calculated by subtracting the non‐encapsulated mRNA concentration from the total mRNA concentration. The encapsulation efficiency was calculated as a percentage using this formula:

$$\mathrm{EE}\;\left(\%\right)=\frac{\mathrm{RNA}\;\mathrm{encapsulated}}{\mathrm{RNA}\;\mathrm{added}\;\mathrm{to}\;\mathrm{assay}}*100$$

### 4.14 Cells, In Vitro Transfection, and Luciferase Assay

HEK293T cells (ATCC, USA) were cultured in complete Dulbecco's modified Eagle's medium (DMEM) (Gibco, ThermoFisher, UK) containing 10% fetal bovine serum (FBS), 1% l‐glutamine and 1% penicillin–streptomycin (Thermo Fisher, UK). Cells were cultured and maintained at 37°C and 5% CO_2_. Cells were plated in a 96‐well plate 24 h prior to transfection to achieve 80% confluency on the day of transfection. Based on the encapsulated RNA concentration measured by RiboGreen, cells were transfected with 100 ng mRNA encapsulated in LNP per well.

Fifty microliters of the cell supernatant were discarded from each well and 50 uL of ONE‐Glo Luciferase (Promega, UK) was added to each well, then placed in the dark. Cells were incubated with the luciferase reagent for 5 min to ensure complete lysis of the cells. Cells were then pipetted up and down and transferred to a white 96‐well Costar plate (ThermoFisher Scientific). The luminescence was then measured on a GloMax Microplate Reader (Promega).

### 4.15 Non‐Lamellar Particle Synthesis and Fusogenicity Studies

Monoolein (glycerol 1‐monooleate; MO; Merck) was prepared as a 14 mM stock solution in ethanol, following previously described procedures [59]. Additional lipid components were substituted for MO within this base formulation, maintaining 14 mM total concentration; DOPE (1,2‐dioleoyl‐sn‐glycero‐3‐phosphoethanolamine, Avanti), DOTAP (1,2‐dioleoyl‐3‐trimethylammonium‐propane, Avanti), DOPC (1,2‐dioleoyl‐sn‐glycero‐3‐phosphocholine, Avanti). Particles were generated using the LipidXplorer platform with the standard microfluidic hydrodynamic focusing (MHF) device (Figure 2a) at a total flow rate of 420 µL min^−^^1^ and a flow‐rate ratio of 1:60 in PBS (pH 7.4). Unless otherwise stated, Pluronic F‐127 (Merck) was included at a final concentration of 0.028 mM to enhance colloidal stability, present during formulation in the aqueous phase.

For fusion assays, DOPC vesicles containing 1 mol% NBD‐PE and 1 mol% Liss Rhodamine‐PE (Avanti) were prepared in bulk from 14 mM ethanol stocks using MHF at a flow rate ratio of 10 in PBS. Vesicle dispersions were subsequently diluted 30‐fold to yield a final lipid concentration of 0.046 mM. Plates containing MO‐based particles produced by LipidXplorer were transferred to a microplate reader (CLARIOstar, BMG Labtech, Germany). Fusion was initiated by injecting 50 µL of the labeled DOPC vesicle solution into each well at 300 µL min^−^^1^. Following excitation at 460 nm, fluorescence emission at 535 and 583 nm was recorded at 1 s intervals over 60 s. Förster resonance energy transfer (FRET) efficiency and the magnitude of FRET change were calculated as described in Equation S6. FRET was normalized to the control composition (100% MO) for comparison between compositional screens.

For stability measurements, MO‐based particles were incubated at room temperature for the specified durations prior to fusion and FRET fluorescence acquisition.

## Author Contributions

B.D. designed and constructed the platform, performed the experiments, and analyzed the data with supervision from Y.E., M.F., and N.B. M.B. assisted with experimental protocols and assay development. M.R.H. and A.A. provided guidance on particle formulations and system design. K.S. directed and performed mRNA synthesis, formulation using the INanoL+ platform, and cell‑transfection experiments, and advised on LNP formulation with the LipidXplorer platform. B.D. wrote the manuscript. N.J.B., M.M.S., D.J.P., M.R.H., R.C., and Y.E. secured the funding. All authors commented on and contributed to the final manuscript. Y.E. supervised the project overall.

## Conflicts of Interest

Molly M. Stevens has invested in, consults for (or is on scientific advisory boards or boards of directors) and conducts sponsored research funded by companies related to the biomaterials field; has filed patent applications related to biomaterials; and has co‐founded companies in the biomaterials field. The rest of the authors declare no conflicts of interests.

## Supporting information




**Supporting File**: adma74165‐sup‐0001‐SuppMat.pdf.

## Data Availability

The data that support the findings of this study are available from the corresponding author upon reasonable request.

## References

[1] X. Hou , T. Zaks , R. Langer , and Y. Dong , “Lipid Nanoparticles for mRNA Delivery,” Nature Reviews Materials 6 (2021): 1078–1094, 10.1038/s41578-021-00358-0. 34394960 PMC8353930

[2] P. Liu , G. Chen , and J. Zhang , “A Review of Liposomes as a Drug Delivery System: Current Status of Approved Products, Regulatory Environments, and Future Perspectives,” Molecules 27 (2022): 1372. 35209162 10.3390/molecules27041372 PMC8879473

[3] H. M. G. Barriga , M. N. Holme , and M. M. Stevens , “Cubosomes: The Next Generation of Smart Lipid Nanoparticles?,” Angewandte Chemie International Edition 58 (2019): 2958–2978, 10.1002/anie.201804067. 29926520 PMC6606436

[4] I. D. Azmi , S. M. Moghimi , and A. Yaghmur , “Cubosomes and Hexosomes as Versatile Platforms for Drug Delivery,” Therapeutic Delivery 6 (2015): 1347–1364, 10.4155/tde.15.81. 26652281

[5] M. Mehta , T. A. Bui , X. Yang , Y. Aksoy , E. M. Goldys , and W. Deng , “Lipid‐Based Nanoparticles for Drug/Gene Delivery: An Overview of the Production Techniques and Difficulties Encountered in Their Industrial Development,” ACS Materials Au 3 (2023): 600–619, 10.1021/acsmaterialsau.3c00032. 38089666 PMC10636777

[6] F. Mazur , M. Bally , B. Stadler , and R. Chandrawati , “Liposomes and Lipid Bilayers in Biosensors,” Advances in Colloid and Interface Science 249 (2017): 88–99, 10.1016/j.cis.2017.05.020. 28602208

[7] Q. Liu and B. J. Boyd , “Liposomes in Biosensors,” Analyst 138 (2013): 391–409, 10.1039/C2AN36140J. 23072757

[8] D. S. Tawfik and A. D. Griffiths , “Man‐Made Cell‐Like Compartments for Molecular Evolution,” Nature Biotechnology 16 (1998): 652–656, 10.1038/nbt0798-652. 9661199

[9] A. S. Ulrich , “Biophysical Aspects of Using Liposomes as Delivery Vehicles,” Bioscience Reports 22 (2002): 129–150, 10.1023/A:1020178304031. 12428898

[10] G. W. Feigenson , “Phase Diagrams and Lipid Domains in Multicomponent Lipid Bilayer Mixtures,” Biochimica et Biophysica Acta (BBA)—Biomembranes 1788 (2009): 47–52, 10.1016/j.bbamem.2008.08.014. 18805392 PMC2637376

[11] S. C. Shetty , N. Yandrapalli , K. Pinkwart , et al., “Directed Signaling Cascades in Monodisperse Artificial Eukaryotic Cells,” ACS Nano 15 (2021): 15656–15666, 10.1021/acsnano.1c04219. 34570489 PMC8552445

[12] C. P. Pilkington , I. Gispert , S. Y. Chui , J. M. Seddon , and Y. Elani , “Engineering a Nanoscale Liposome‐in‐Liposome for In Situ Biochemical Synthesis and Multi‐Stage Release,” Nature Chemistry 16 (2024): 1612–1620, 10.1038/s41557-024-01584-z. PMC11446840 39009794

[13] K. P. Adamala , D. A. Martin‐Alarcon , K. R. Guthrie‐Honea , and E. S. Boyden , “Engineering Genetic Circuit Interactions Within and Between Synthetic Minimal Cells,” Nature Chemistry 9 (2017): 431–439, 10.1038/nchem.2644. PMC5407321 28430194

[14] S. S. Mansy , J. P. Schrum , M. Krishnamurthy , S. Tobé , D. A. Treco , and J. W. Szostak , “Template‐Directed Synthesis of a Genetic Polymer in a Model Protocell,” Nature 454 (2008): 122–125, 10.1038/nature07018. 18528332 PMC2743009

[15] C. Meyer , A. Arizzi , T. Henson , et al., “Designer Artificial Environments for Membrane Protein Synthesis,” Nature Communications 16 (2025): 4363, 10.1038/s41467-025-59471-1. PMC12065789 40348791

[16] C. Dietrich , L. A. Bagatolli , Z. N. Volovyk , et al., “Lipid Rafts Reconstituted in Model Membranes,” Biophysical Journal 80 (2001): 1417–1428, 10.1016/S0006-3495(01)76114-0. 11222302 PMC1301333

[17] P. R. Cullis and M. J. Hope , “Effects of Fusogenic Agent on Membrane Structure of Erythrocyte Ghosts and the Mechanism of Membrane Fusion,” Nature 271 (1978): 672–674, 10.1038/271672a0. 625336

[18] J. Philipp , A. Dabkowska , A. Reiser , et al., “pH‐Dependent Structural Transitions in Cationic Ionizable Lipid Mesophases are Critical for Lipid Nanoparticle Function,” Proceedings of the National Academy of Sciences U S A 120 (2023): 2310491120, 10.1073/pnas.2310491120. PMC10723131 38055742

[19] A. G. Lee , “How Lipids Affect the Activities of Integral Membrane Proteins,” Biochimica et Biophysica Acta (BBA)—Biomembranes 1666 (2004): 62–87, 10.1016/j.bbamem.2004.05.012. 15519309

[20] D. Marsh , “Protein Modulation of Lipids, and Vice‐Versa, in Membranes,” Biochimica et Biophysica Acta (BBA)—Biomembranes 1778 (2008): 1545–1575, 10.1016/j.bbamem.2008.01.015. 18294954

[21] A. Angelopoulos , J. F. Cahoon , and R. Alterovitz , “Transforming Science Labs into Automated Factories of Discovery,” Science Robotics 9 (2024): adm6991, 10.1126/scirobotics.adm6991. 39441898

[22] W. E. Arter , R. Qi , N. A. Erkamp , et al., “Biomolecular Condensate Phase Diagrams With a Combinatorial Microdroplet Platform,” Nature Communications 13 (2022): 7845, 10.1038/s41467-022-35265-7. PMC9768726 36543777

[23] S. A. H. Jansen , L. S. A. Dreyer , J. van Basten , et al., “PhaseXplorer Creates High‐Dimensional Phase Diagrams With Closed‐Loop Active Learning,” ACS Nano 19 (2025): 38981–38989, 10.1021/acsnano.5c07268. 41182865 PMC12632172

[24] M. Fletcher , B. Diggines , and Y. Elani , “Molecular Systems Engineering of Synthetic Cells,” Nature Chemistry 18 (2026): 14–22, 10.1038/s41557-025-02019-z. 41495211

[25] B. J. Frisken , C. Asman , and P. J. Patty , “Studies of Vesicle Extrusion,” Langmuir 16 (2000): 928–933, 10.1021/la9905113.

[26] L. Cui , S. Pereira , S. Sonzini , et al., “Development of a High‐Throughput Platform for Screening Lipid Nanoparticles for mRNA Delivery,” Nanoscale 14 (2022): 1480–1491, 10.1039/D1NR06858J. 35024714

[27] C. Zhu , N. Roa , E. Neathery , et al., “Comparative Technical and Operational Assessment of Current and Emerging Bench‐Scale Lipid Nanoparticle Platforms for Production of mRNA Vaccines,” Vaccine: X 28 (2026): 100771.

[28] U. Labs, Sunscreen, https://www.unchainedlabs.com/sunscreen/, (2025).

[29] A. R. Hanna , S. J. Shepherd , G. A. Datto , et al., “Automated and Parallelized Microfluidic Generation of Large and Precisely Defined Lipid Nanoparticle Libraries,” ACS Nano 20 (2026): 772–789, 10.1021/acsnano.5c15613. 41453807

[30] C. S. Hansel , D. L. Plant , G. A. Holdgate , M. J. Collier , and H. Plant , “Advancing Automation in High‐Throughput Screening: Modular Unguarded Systems Enable Adaptable Drug Discovery,” Drug Discovery Today 27 (2022): 2051–2056, 10.1016/j.drudis.2022.03.010. 35304338

[31] K. Dauer , S. Pfeiffer‐Marek , W. Kamm , and K. G. Wagner , “Microwell Plate‐Based Dynamic Light Scattering as a High‐Throughput Characterization Tool in Biopharmaceutical Development,” Pharmaceutics 13 (2021): 172. 33514069 10.3390/pharmaceutics13020172 PMC7911513

[32] M. Fletcher and Y. Elani , “On‐the‐Fly Microfluidic Control of Giant Vesicle Compositions Validated by DNA Surface Charge Sensors,” ACS Nano 19 (2025): 13768–13778, 10.1021/acsnano.4c16289. 40183490 PMC12004935

[33] A. Jahn , W. N. Vreeland , M. Gaitan , and L. E. Locascio , “Controlled Vesicle Self‐Assembly in Microfluidic Channels With Hydrodynamic Focusing,” Journal of the American Chemical Society 126 (2004): 2674–2675, 10.1021/ja0318030. 14995164

[34] A. Jahn , S. M. Stavis , J. S. Hong , W. N. Vreeland , D. L. DeVoe , and M. Gaitan , “Microfluidic Mixing and the Formation of Nanoscale Lipid Vesicles,” ACS Nano 4 (2010): 2077–2087, 10.1021/nn901676x. 20356060

[35] J. Huang , J. T. Buboltz , and G. W. Feigenson , “Maximum Solubility of Cholesterol in Phosphatidylcholine and Phosphatidylethanolamine Bilayers,” Biochimica et Biophysica Acta (BBA)—Biomembranes 1417 (1999): 89–100, 10.1016/S0005-2736(98)00260-0. 10076038

[36] L. F. Aguilar , J. A. Pino , M. A. Soto‐Arriaza , F. J. Cuevas , S. Sanchez , and C. P. Sotomayor , “Differential Dynamic and Structural Behavior of Lipid–Cholesterol Domains in Model Membranes,” PLoS One 7 (2012): 40254, 10.1371/journal.pone.0040254. PMC3386959 22768264

[37] P. Carravilla , J. L. Nieva , F. M. Goni , J. Requejo‐Isidro , and N. Huarte , “Two‐Photon Laurdan Studies of the Ternary Lipid Mixture DOPC:SM:Cholesterol Reveal a Single Liquid Phase at Sphingomyelin:Cholesterol Ratios Lower than 1,” Langmuir 31 (2015): 2808–2817, 10.1021/la504251u. 25658036

[38] L. D. Mayer , M. J. Hope , and P. R. Cullis , “Vesicles of Variable Sizes Produced by a Rapid Extrusion Procedure,” Biochimica et Biophysica Acta (BBA)—Biomembranes 858 (1986): 161–168, 10.1016/0005-2736(86)90302-0. 3707960

[39] L. F. Aguilar , J. A. Pino , M. A. Soto‐Arriaza , F. J. Cuevas , S. Sánchez , and C. P. Sotomayor , “Differential Dynamic and Structural Behavior of Lipid–Cholesterol Domains in Model Membranes,” PLoS One 7 (2012): 40254, 10.1371/journal.pone.0040254. PMC3386959 22768264

[40] S. L. Veatch and S. L. Keller , “Separation of Liquid Phases in Giant Vesicles of Ternary Mixtures of Phospholipids and Cholesterol,” Biophysical Journal 85 (2003): 3074–3083, 10.1016/S0006-3495(03)74726-2. 14581208 PMC1303584

[41] A. Mangiarotti , E. Sabri , K. V. Schmidt , et al., “Lipid Packing and Cholesterol Content Regulate Membrane Wetting and Remodeling by Biomolecular Condensates,” Nature Communications 16 (2025): 2756, 10.1038/s41467-025-57985-2. PMC11926106 40113768

[42] I. Schachter , R. O. Paananen , B. Fábián , P. Jurkiewicz , and M. Javanainen , “The Two Faces of the Liquid Ordered Phase,” Journal of Physical Chemistry Letters 13 (2022): 1307–1313, 10.1021/acs.jpclett.1c03712. 35104407 PMC8842317

[43] A. Mangiarotti , M. Siri , N. W. Tam , Z. Zhao , L. Malacrida , and R. Dimova , “Biomolecular Condensates Modulate Membrane Lipid Packing and Hydration,” Nature Communications 14 (2023): 6081, 10.1038/s41467-023-41709-5. PMC10539446 37770422

[44] L. B. P. Socas , J. A. Valdivia‐Perez , M. L. Fanani , and E. E. Ambroggio , “Multidimensional Spectral Phasors of LAURDAN's Excitation–Emission Matrices: The Ultimate Sensor for Lipid Phases?,” Journal of the American Chemical Society 146 (2024): 17230–17239, 10.1021/jacs.4c03443. 38874760

[45] A. Mangiarotti and R. Dimova , “The Spectral Phasor Approach to Resolving Membrane Order With Environmentally Sensitive Dyes,” Methods in Enzymology 700 (2024): 105–126. 38971597 10.1016/bs.mie.2024.01.024

[46] R. Ziblat , L. Leiserowitz , and L. Addadi , “Crystalline Domain Structure and Cholesterol Crystal Nucleation in Single Hydrated DPPC:Cholesterol:POPC Bilayers,” Journal of the American Chemical Society 132 (2010): 9920–9927, 10.1021/ja103975g. 20586463

[47] T. G. Meikle , C. E. Conn , F. Separovic , and C. J. Drummond , “Exploring the Structural Relationship Between Encapsulated Antimicrobial Peptides and the Bilayer Membrane Mimetic Lipidic Cubic Phase: Studies With Gramicidin A′,” RSC Advances 6 (2016): 68685–68694, 10.1039/C6RA13658C.

[48] K. J. Hassett , K. E. Benenato , E. Jacquinet , et al., “Optimization of Lipid Nanoparticles for Intramuscular Administration of mRNA Vaccines,” Molecular Therapy—Nucleic Acids 15 (2019): 1–11, 10.1016/j.omtn.2019.01.013. 30785039 PMC6383180

[49] N. M. Belliveau , J. Huft , P. J. Lin , et al., “Microfluidic Synthesis of Highly Potent Limit‐Size Lipid Nanoparticles for In Vivo Delivery of siRNA,” Molecular Therapy—Nucleic Acids 1 (2012): 37. 10.1038/mtna.2012.28 PMC3442367 23344179

[50] N. Kimura , M. Maeki , Y. Sato , et al., “Development of the iLiNP Device: Fine Tuning the Lipid Nanoparticle Size Within 10 Nm for Drug Delivery,” ACS Omega 3 (2018): 5044–5051, 10.1021/acsomega.8b00341. 31458718 PMC6641893

[51] S. C. Semple , A. Akinc , J. Chen , et al., “Rational Design of Cationic Lipids for siRNA Delivery,” Nature Biotechnology 28 (2010): 172–176, 10.1038/nbt.1602. 20081866

[52] A. G. Fedorovskiy , D. N. Antropov , A. S. Dome , et al., “Novel Efficient Lipid‐Based Delivery Systems Enable a Delayed Uptake and Sustained Expression of mRNA in Human Cells and Mouse Tissues,” Pharmaceutics 16 (2024): 684. 38794346 10.3390/pharmaceutics16050684 PMC11125954

[53] I. M. Hafez and P. R. Cullis , “Roles of Lipid Polymorphism in Intracellular Delivery,” Advanced Drug Delivery Reviews 47 (2001): 139–148, 10.1016/S0169-409X(01)00103-X. 11311989

[54] I. Koltover , T. Salditt , J. O. Rädler , and C. R. Safinya , “An Inverted Hexagonal Phase of Cationic Liposome‐DNA Complexes Related to DNA Release and Delivery,” Science 281 (1998): 78–81, 10.1126/science.281.5373.78. 9651248

[55] G. Sahay , W. Querbes , C. Alabi , et al., “Efficiency of siRNA Delivery by Lipid Nanoparticles is Limited by Endocytic Recycling,” Nature Biotechnology 31 (2013): 653–658, 10.1038/nbt.2614. PMC3814166 23792629

[56] A. Jain , S. P. Ong , G. Hautier , et al., “Commentary: The Materials Project: A Materials Genome Approach to Accelerating Materials Innovation,” APL Materials 1 (2013): 011002.

[57] P. R. Cullis , M. J. Hope , and C. P. S. Tilcock , “Lipid Polymorphism and the Roles of Lipids in Membranes,” Chemistry and Physics of Lipids 40 (1986): 127–144, 10.1016/0009-3084(86)90067-8. 3742670

[58] J. Zhai , C. Fong , N. Tran , and C. J. Drummond , “Non‐Lamellar Lyotropic Liquid Crystalline Lipid Nanoparticles for the Next Generation of Nanomedicine,” ACS Nano 13 (2019): 6178–6206, 10.1021/acsnano.8b07961. 31082192

[59] C. P. Pilkington , C. Contini , J. D. Barritt , P. A. Simpson , J. M. Seddon , and Y. Elani , “A Microfluidic Platform for the Controlled Synthesis of Architecturally Complex Liquid Crystalline Nanoparticles,” Scientific Reports 13 (2023): 12684, 10.1038/s41598-023-39205-3. 37542147 PMC10403506

[60] P. Stępień , S. Świątek , M. Y. Y. Robles , et al., “CRAFTing Delivery of Membrane Proteins into Protocells Using Nanodiscs,” ACS Applied Materials & Interfaces 15 (2023): 56689–56701. 38015973 10.1021/acsami.3c11894 PMC10726305

[61] D. K. Struck , D. Hoekstra , and R. E. Pagano , “Use of Resonance Energy Transfer to Monitor Membrane Fusion,” Biochemistry 20 (1981): 4093–4099, 10.1021/bi00517a023. 7284312

[62] G. Son , J. Song , J. C. Park , H. N. Kim , and H. Kim , “Fusogenic Lipid Nanoparticles for Rapid Delivery of Large Therapeutic Molecules to Exosomes,” Nature Communications 16 (2025): 4799, 10.1038/s41467-025-59489-5. PMC12102247 40410169

[63] D. P. Siegel , W. J. Green , and Y. Talmon , “The Mechanism of Lamellar‐to‐Inverted Hexagonal Phase Transitions: A Study Using Temperature‐Jump Cryo‐Electron Microscopy,” Biophysical Journal 66 (1994): 402–414, 10.1016/S0006-3495(94)80790-8. 8161694 PMC1275708

[64] C. R. Safinya , K. K. Ewert , R. N. Majzoub , and C. Leal , “Cationic Liposome–Nucleic Acid Complexes for Gene Delivery and Gene Silencing,” New Journal of Chemistry 38 (2014): 5164–5172, 10.1039/C4NJ01314J. 25587216 PMC4288823

[65] A. Joardar , G. P. Pattnaik , and H. Chakraborty , “Effect of Phosphatidylethanolamine and Oleic Acid on Membrane Fusion: Phosphatidylethanolamine Circumvents the Classical Stalk Model,” Journal of Physical Chemistry B 125 (2021): 13192–13202, 10.1021/acs.jpcb.1c08044. 34839659

[66] A. J. Tilley , C. J. Drummond , and B. J. Boyd , “Disposition and Association of the Steric Stabilizer Pluronic® F127 in Lyotropic Liquid Crystalline Nanostructured Particle Dispersions,” Journal of Colloid and Interface Science 392 (2013): 288–296, 10.1016/j.jcis.2012.09.051. 23137909

[67] P. T. Spicer , K. L. Hayden , M. L. Lynch , A. Ofori‐Boateng , and J. L. Burns , “Novel Process for Producing Cubic Liquid Crystalline Nanoparticles (Cubosomes),” Langmuir 17 (2001): 5748–5756, 10.1021/la010161w.

[68] B. Angelov , A. Angelova , U. Vainio , et al., “Long‐Living Intermediates During a Lamellar to a Diamond‐Cubic Lipid Phase Transition: A Small‐Angle X‐Ray Scattering Investigation,” Langmuir 25 (2009): 3734–3742, 10.1021/la804225j. 19708151

[69] G. Wörle , M. Drechsler , M. H. J. Koch , B. Siekmann , K. Westesen , and H. Bunjes , “Influence of Composition and Preparation Parameters on the Properties of Aqueous Monoolein Dispersions,” International Journal of Pharmaceutics 329 (2007): 150–157. 16987623 10.1016/j.ijpharm.2006.08.023

