# Supplementary material for: Navigating Lipid Nanostructure Design Space Through Continuous Microfluidic Automation
Source: Adv Mater. 2026 Jul 27;38(52):e74165. doi: 10.1002/adma.74165 (PMC13579175; doi:10.1002/adma.74165)
Supplement: Supplementary file 1 [file ADMA-38-e74165-s001.pdf]

Supplementary Information

$$f_x = \frac{FR_x C_x}{\sum_{i=1}^{N}(FR_i C_i)}$$

Supplementary Eq. 1 – Fractional composition f_x of a lipid component x, where FR_i is the flow-rate ratio for stream i, C_i is the initial concentration of the relevant component in stream i, and N is the total number of streams.

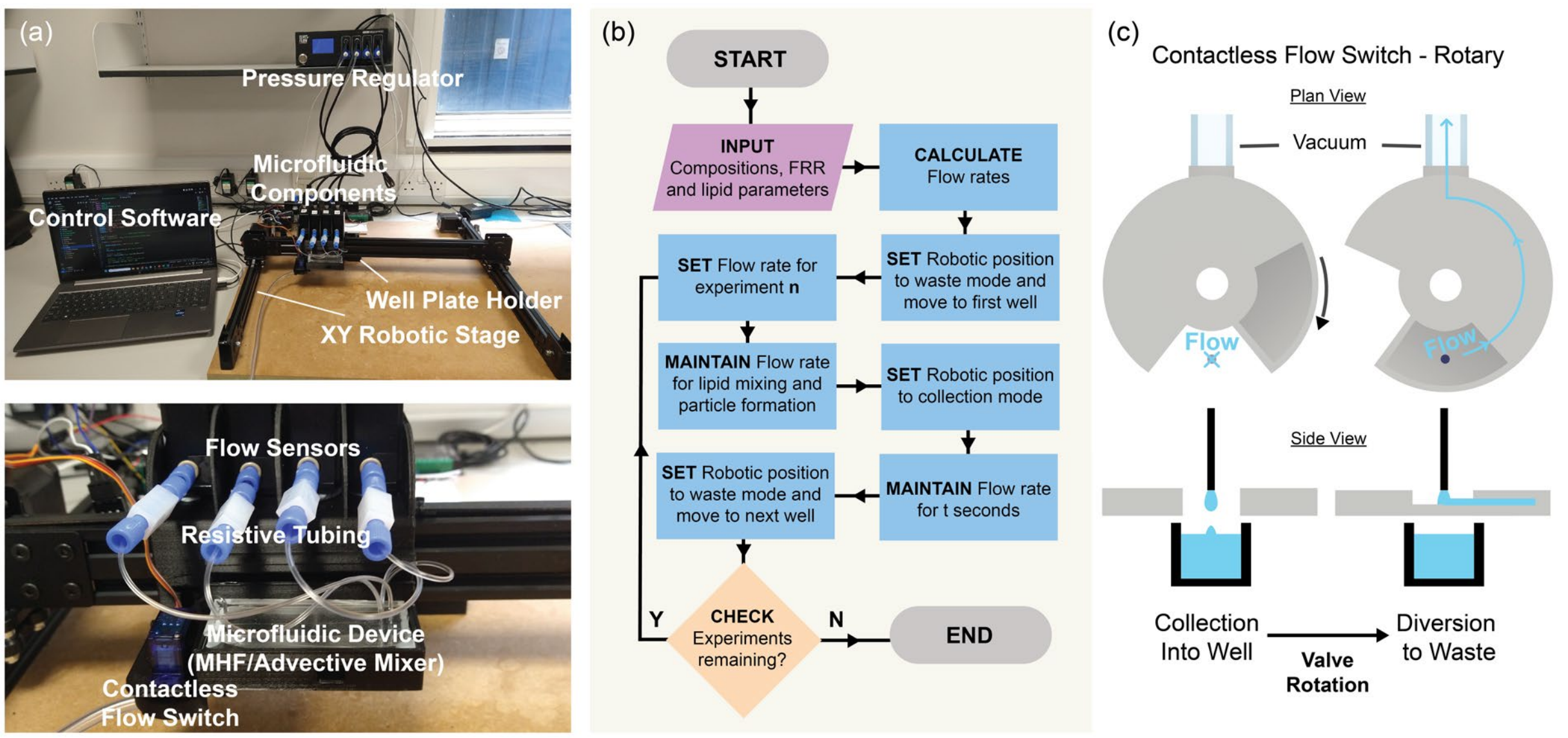

Supplementary Fig. 1 – Platform Concepts. **(a)** Image of LipidXplorer setup labelled with key components. **(b)** System control flow chart for the series of operations for producing and collecting a given sample. **(c)** Contactless control valve to direct flow towards well position/waste without disrupting on-chip flow. The valve actuates by rotating around its central axis. This either allows the flow to expel into the aligned well or diverts towards a vacuum-driven waste line. Given the chip outlet remains at atmospheric pressure at all states, switching does not affect the continuous flow profile on chip.

$$FR_{error} = FR_{target} - FR_{measured}$$

PI CORRECTION $\quad \Delta P = K_p abs(FR_{error}) FR_{error} + K_i \int F_{error}\, dt$

INTERPOLATION $\quad IF\ FR_{err(t-1)} \times FR_{errt} < 0: \quad \Delta P = P_t + \frac{P_t - P_{t-1}}{FR_t - FR_{t-1}} \times (FR_{error})$

Supplementary Eq. 2 – Custom algorithm to determine pressure change (ΔP), given the flow rate error (FR_{error}), derived from the target flow rate (FR_{target}) and the sensor-measured flow rate ($FR_{measured}$) for each cycle. K_p and K_i are PI parameters and P_t is the pressure at the current timepoint. The timepoint is interval between cycles of the PI loop, set to 0.5s as standard. Interpolation is performed if the two preceding points lie on either side of the setpoint. This addition helps to dampen oscillation caused by strong PI parameters, allowing fast flow rate changes while maintaining high stability once the set-point has been reached.

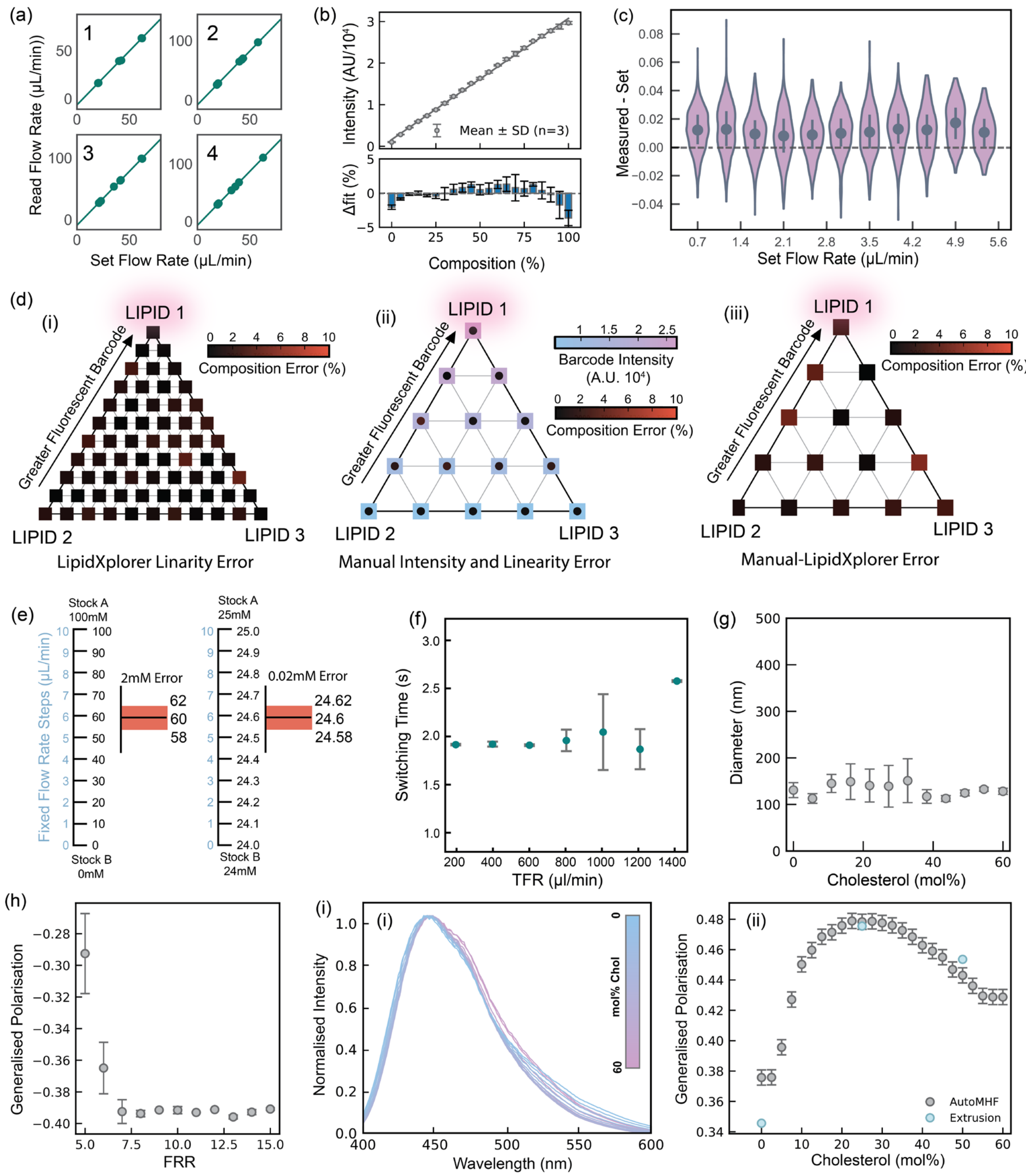

Supplementary Fig. 2 – Validation of Performance **(a)** Flow sensor calibrations achieved via gravimetric measurements **(b)** Fluorescent barcoding of labelled nanoparticles produced at variable compositions with LipidXplorer, and deviation from ideal linearity. Most prominent deviation at edges corresponding to 0 flow condition. **(c)** Flow rate error between measured (sensor reading) and set flow rates for several set flow rate values n=6891 over all conditions. **(d)** Quantification of errors over ternary composition space derived from the intensity of fluorescently labelled lipid stock. Labelled DOPC mixing with unlabelled DOPC stocks (i) Compositional errors for particle compositions produced by the LipidXplorer platform extracted as deviation from linearity along fixed compositional steps. (ii) Intensities and linearity errors for particles manually prepared via pipetting for 25% increments over the same space (iii) calculated compositional error between the manual and LipidXplorer prepared compositions **(e)** Schematic illustration of how the same relative flow-control error produces different absolute concentration uncertainties depending on the stock concentration range. Interpolating between broadly spaced stocks produces a larger absolute uncertainty, whereas stocks with a closer interval reduce the flow-control-derived concentration uncertainty. This schematic isolates the flow-control-derived uncertainty; additional experimental errors may also contribute to the total concentration uncertainty. **(f)** Switching time, the time between setting a new flow

rate condition and reaching equilibrium stability at various TFRs. Switching time remains below 3s in all conditions but becomes more variable at higher TFRs. **(g)** Diameter of DOPC particles with increasing cholesterol as determined by DLS. Decrease in size distribution above 40% may be due to a structural transition but this will need be verified via X-ray diffraction in future work **(h)** Effect of flow rate ratio on the generalised polarisation of the membrane probe Laurdan, a function of ethanol concentration. Minimal effect is seen at flow rate ratios above 10. **(i)** Effect of cholesterol additions to DPPC on Laurdan properties in samples produced by LipidXplorer (i-i) Laurdan spectra shows minimal difference with increasing cholesterol. (i-ii) The small changes in GP that occurs is quantified by generalised polarisation, and matches with samples produced manually via extrusion.

$$V = \frac{TFR}{60} \cdot 2.5 + 12$$

Supplementary Eq. 3 – Waste volume per samples (V_w) in μL, where the total flow rate (TFR) is in μL/min. Fixed addition of 12 μL related to the dead volume through tubing.

$$t = \frac{V_c + 12}{TFR} + t_s$$

Supplementary Eq. 4 – Formulation time per sample (s) for a given collection volume (V_c), total flow rate (TFR) and switching time (t_s)

$$GP = \frac{I_{435\pm2} - I_{490\pm2}}{I_{435\pm2} + I_{490\pm2}}$$

Supplementary Eq. 5 – Generalised polarisation of Laurdan emission. Ratios of intensity taken at the two characteristic peaks.

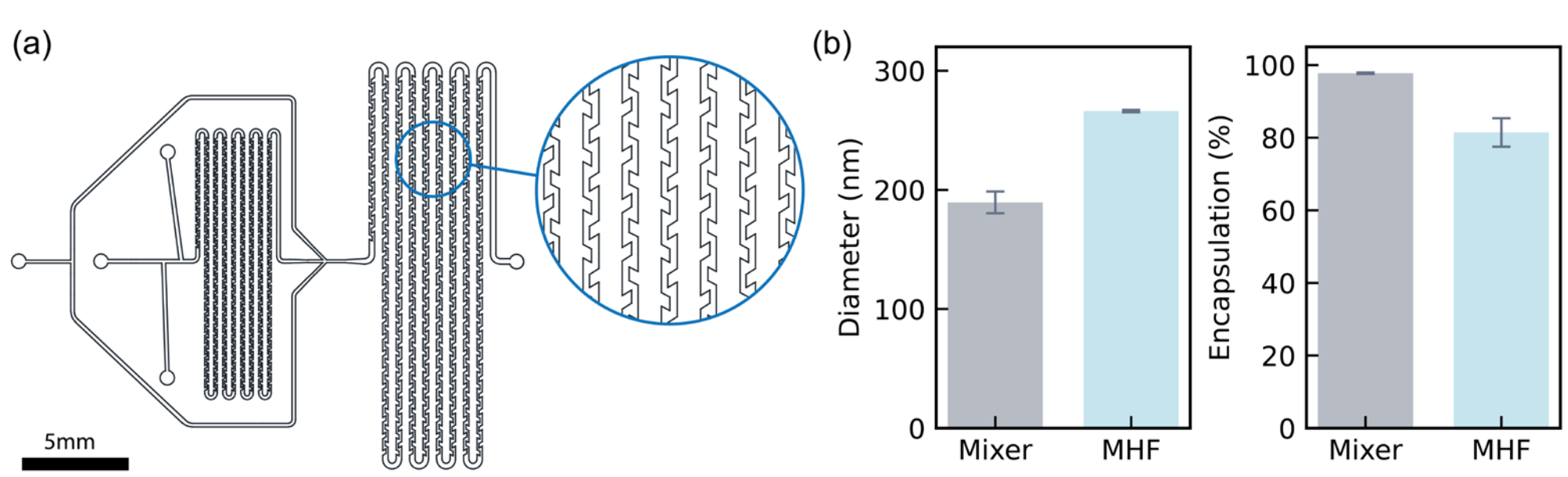

Supplementary Fig. 3 – mRNA LNP Formulation **(a)** Device geometry adapted to include advective mixing baffles post the mixing junction to improve encapsulation. **(b)** Comparison of advective mixer and MHF chip geometries for the formulation of SM102 based LNPs, demonstrating the mixer can achieve smaller particles with higher encapsulation (mean ± SD (n = 3)).

$$E(t) = \frac{I_{Acceptor}(t)}{I_{Acceptor}(t) + I_{Donor}(t)}$$

$$|\Delta E_{\%}| = \left|\frac{E_{tmax} - E_0}{E_0}\right|$$

Supplementary Eq. 6 – FRET Equations. (a) FRET efficiency (E) at time t, given the intensities of acceptor (583nm) and donor (535nm). (b) Change in FRET (%) between t_{max} and t_0 (the initial timepoint).

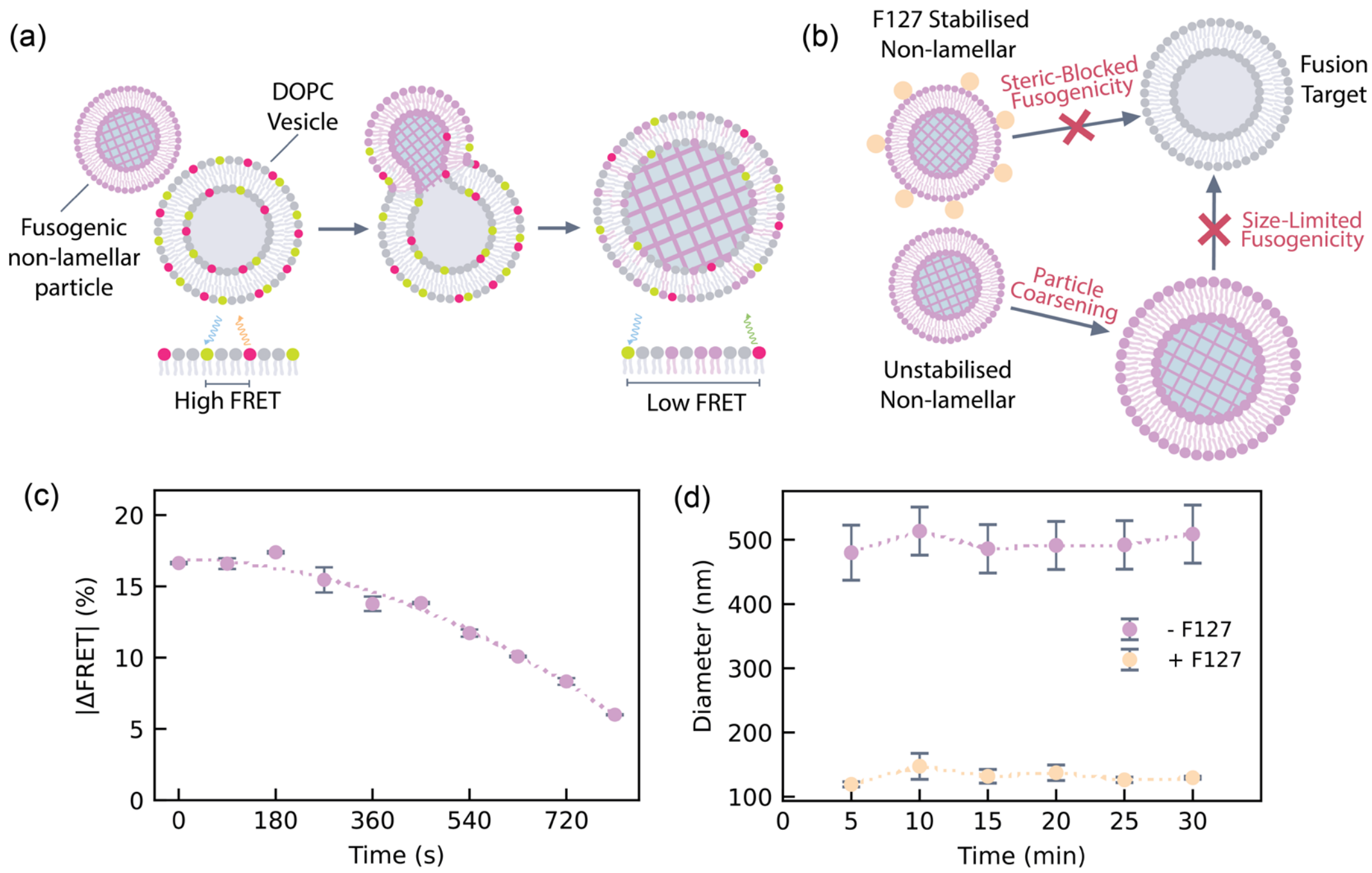

Supplementary Fig. 4 – Non-lamellar particle fusion **(a)** Detailed schematic of Förster resonance energy transfer dilution assay. Fluorophore rich DOPC target vesicle undergoes fusion with non-lamellar particle resulting in dilution of fluorophores and decrease in FRET signal **(b)** Schematic illustrating the proposed effects of Pluronic stabilisation: F-127 acts as a steric barrier to membrane fusion, while its absence permits particle coarsening beyond the curvature regime required for efficient fusion. **(c)** Fusogenicity of unstabilised (no F127) monoolein nanoparticles at a given timepoint post formulation. **(d)** Size comparison of MO particles with and without F-127 stabilising polymer. Unstabilised particles are observed to a have significantly increased (~4-5x) hydrodynamic diameter compared to those stabilised. In both cases, the diameter remains largely constant over the 30 min observation period.
